# Author Correction: Landscape of multi-nucleotide variants in 125,748 human exomes and 15,708 genomes

**DOI:** 10.1038/s41467-021-21077-8

**Published:** 2021-02-02

**Authors:** Qingbo Wang, Emma Pierce-Hoffman, Beryl B. Cummings, Jessica Alföldi, Laurent C. Francioli, Laura D. Gauthier, Andrew J. Hill, Anne H. O’Donnell-Luria, Genome Aggregation Database Production Team, Irina M. Armean, Eric Banks, Louis Bergelson, Kristian Cibulskis, Ryan L. Collins, Kristen M. Connolly, Miguel Covarrubias, Mark J. Daly, Stacey Donnelly, Yossi Farjoun, Steven Ferriera, Stacey Gabriel, Jeff Gentry, Namrata Gupta, Thibault Jeandet, Diane Kaplan, Kristen M. Laricchia, Christopher Llanwarne, Eric V. Minikel, Ruchi Munshi, Benjamin M. Neale, Sam Novod, Nikelle Petrillo, Timothy Poterba, David Roazen, Valentin Ruano-Rubio, Andrea Saltzman, Kaitlin E. Samocha, Molly Schleicher, Cotton Seed, Matthew Solomonson, Jose Soto, Grace Tiao, Kathleen Tibbetts, Charlotte Tolonen, Christopher Vittal, Gordon Wade, Arcturus Wang, James S. Ware, Nicholas A. Watts, Ben Weisburd, Nicola Whiffin, Genome Aggregation Database Consortium, Carlos A. Aguilar Salinas, Tariq Ahmad, Christine M. Albert, Diego Ardissino, Gil Atzmon, John Barnard, Laurent Beaugerie, Emelia J. Benjamin, Michael Boehnke, Lori L. Bonnycastle, Erwin P. Bottinger, Donald W. Bowden, Matthew J. Bown, John C. Chambers, Juliana C. Chan, Daniel Chasman, Judy Cho, Mina K. Chung, Bruce Cohen, Adolfo Correa, Dana Dabelea, Dawood Darbar, Ravindranath Duggirala, Josée Dupuis, Patrick T. Ellinor, Roberto Elosua, Jeanette Erdmann, Tõnu Esko, Martti Färkkilä, Jose Florez, Andre Franke, Gad Getz, Benjamin Glaser, Stephen J. Glatt, David Goldstein, Clicerio Gonzalez, Leif Groop, Christopher Haiman, Craig Hanis, Matthew Harms, Mikko Hiltunen, Matti M. Holi, Christina M. Hultman, Mikko Kallela, Jaakko Kaprio, Sekar Kathiresan, Bong-Jo Kim, Young Jin Kim, George Kirov, Jaspal Kooner, Seppo Koskinen, Harlan M. Krumholz, Subra Kugathasan, Soo Heon Kwak, Markku Laakso, Terho Lehtimäki, Ruth J. F. Loos, Steven A. Lubitz, Ronald C. W. Ma, Jaume Marrugat, Kari M. Mattila, Steven McCarroll, Mark I. McCarthy, Dermot McGovern, Ruth McPherson, James B. Meigs, Olle Melander, Andres Metspalu, Peter M. Nilsson, Michael C. O’Donovan, Dost Ongur, Lorena Orozco, Michael J. Owen, Colin N. A. Palmer, Aarno Palotie, Kyong Soo Park, Carlos Pato, Ann E. Pulver, Nazneen Rahman, Anne M. Remes, John D. Rioux, Samuli Ripatti, Dan M. Roden, Danish Saleheen, Veikko Salomaa, Nilesh J. Samani, Jeremiah Scharf, Heribert Schunkert, Moore B. Shoemaker, Pamela Sklar, Hilkka Soininen, Harry Sokol, Tim Spector, Patrick F. Sullivan, Jaana Suvisaari, E. Shyong Tai, Yik Ying Teo, Tuomi Tiinamaija, Ming Tsuang, Dan Turner, Teresa Tusie-Luna, Erkki Vartiainen, Hugh Watkins, Rinse K. Weersma, Maija Wessman, James G. Wilson, Ramnik J. Xavier, Marquis P. Vawter, Konrad J. Karczewski, Daniel G. MacArthur

**Affiliations:** 1 Program in Medical and Population Genetics, The Broad Institute of MIT and Harvard, Cambridge, MA 02142 USA; 2 Analytic and Translational Genetics Unit, Massachusetts General Hospital, Boston, MA 02114 USA; 3 Program in Bioinformatics and Integrative Genomics, Harvard Medical School, Boston, MA 02115 USA; 4 Program in Biomedical and Biological Sciences, Harvard Medical School, Boston, MA 02115 USA; 5 Data Sciences Platform, Broad Institute of MIT and Harvard, Cambridge, MA 02142 USA; 6 Department of Genome Sciences, University of Washington, Seattle, WA 98195 USA; 7 Centre for Population Genomics, Garvan Institute of Medical Research, and UNSW Sydney, Sydney, Australia; 8 Centre for Population Genomics, Murdoch Children’s Research Institute, Melbourne, Australia; 9 European Molecular Biology Laboratory, European Bioinformatics Institute, Wellcome Genome Campus, Hinxton, Cambridge, CB10 1SD UK; 10 Center for Genomic Medicine, Massachusetts General Hospital, Boston, MA 02114 USA; 11 Genomics Platform, Broad Institute of MIT and Harvard, Cambridge, MA 02142 USA; 12 Stanley Center for Psychiatric Research, Broad Institute of MIT and Harvard, Cambridge, MA 02142 USA; 13 Broad Genomics, Broad Institute of MIT and Harvard, Cambridge, MA 02142 USA; 14 Wellcome Sanger Institute, Wellcome Genome Campus, Hinxton, Cambridge, CB10 1SA UK; 15 National Heart & Lung Institute and MRC London Institute of Medical Sciences, Imperial College London, London, W12 0NN UK; 16 Cardiovascular Research Centre, Royal Brompton & Harefield Hospitals NHS Trust, London, SW3 6NP UK; 17 Unidad de Investigacion de Enfermedades Metabolicas. Instituto Nacional de Ciencias Medicas y Nutricion, Mexico City, 14080 Mexico; 18 Peninsula College of Medicine and Dentistry, Exeter, EX25DW UK; 19 Division of Preventive Medicine, Brigham and Women’s Hospital, Boston, MA 02115 USA; 20 Division of Cardiovascular Medicine, Brigham and Women’s Hospital and Harvard Medical School, Boston, MA 02115 USA; 21 Department of Cardiology, University Hospital, 43100 Parma, Italy; 22 Department of Biology, Faculty of Natural Sciences, University of Haifa, Haifa, 3498838 Israel; 23 Departments of Medicine and Genetics, Albert Einstein College of Medicine, Bronx, NY 10461 USA; 24 Department of Quantitative Health Sciences, Lerner Research Institute, Cleveland Clinic, Cleveland, OH 44122 USA; 25 Sorbonne Université, APHP, Gastroenterology Department, Saint Antoine Hospital, Paris, 75012 France; 26 NHLBI and Boston University’s Framingham Heart Study, Framingham, MA 01702 USA; 27 Department of Medicine, Boston University School of Medicine, Boston, MA 02118 USA; 28 Department of Epidemiology, Boston University School of Public Health, Boston, MA 02118 USA; 29 Department of Biostatistics and Center for Statistical Genetics, University of Michigan, Ann Arbor, MI 48109 USA; 30 National Human Genome Research Institute, National Institutes of Health, Bethesda, MD 20892 USA; 31 The Charles Bronfman Institute for Personalized Medicine, Icahn School of Medicine at Mount Sinai, New York, NY 10029 USA; 32 Department of Biochemistry, Wake Forest School of Medicine, Winston-Salem, NC 27101 USA; 33 Center for Genomics and Personalized Medicine Research, Wake Forest School of Medicine, Winston-Salem, NC 27157 USA; 34 Center for Diabetes Research, Wake Forest School of Medicine, Winston-Salem, NC 27101 USA; 35 Department of Cardiovascular Sciences, University of Leicester, Leicester, LE1 7RH UK; 36 NIHR Leicester Biomedical Research Centre, Glenfield Hospital, Leicester, LE3 9QP UK; 37 Department of Epidemiology and Biostatistics, Imperial College London, London, W2 1PG UK; 38 Department of Cardiology, Ealing Hospital NHS Trust, Southall, UB1 3HW UK; 39 Imperial College Healthcare NHS Trust, Imperial College London, London, W2 1NY UK; 40 Department of Medicine and Therapeutics, The Chinese University of Hong Kong, Hong Kong, China; 41 Department of Medicine, Harvard Medical School, Boston, MA 02115 USA; 42 Departments of Cardiovascular Medicine, Cellular and Molecular Medicine, Molecular Cardiology and Quantitative Health Sciences, Cleveland Clinic, Cleveland, OH 44195 USA; 43 McLean Hospital, Belmont, MA 02478 USA; 44 Department of Medicine, University of Mississippi Medical Center, Jackson, MS 39216 USA; 45 Department of Epidemiology, Colorado School of Public Health, Aurora, CO 80045 USA; 46 Department of Medicine and Pharmacology, University of Illinois at Chicago, Chicago, IL 60612 USA; 47 Department of Genetics, Texas Biomedical Research Institute, San Antonio, TX 78227 USA; 48 Department of Biostatistics, Boston University School of Public Health, Boston, MA 02118 USA; 49 Cardiac Arrhythmia Service and Cardiovascular Research Center, Massachusetts General Hospital, Boston, MA 02114 USA; 50 Cardiovascular Epidemiology and Genetics, Hospital del Mar Medical Research Institute (IMIM), Barcelona, 08003 Catalonia Spain; 51 CIBER CV, Barcelona, 08017 Catalonia Spain; 52 Department of Medicine, Medical School, University of Vic-Central University of Catalonia, Barcelona, 08500 Spain; 53 Institute for Cardiogenetics, University of Lübeck, Lübeck, 23562 Germany; 54 DZHK (German Research Centre for Cardiovascular Research), Partner Site Hamburg/Lübeck/Kiel, 23562 Lübeck, Germany; 55 University Heart Center Lübeck, 23562 Lübeck, Germany; 56 Estonian Genome Center, Institute of Genomics, University of Tartu, Tartu, 51003 Estonia; 57 Helsinki University and Helsinki University Hospital, Clinic of Gastroenterology, Helsinki, 00100 Finland; 58 Institute of Clinical Molecular Biology (IKMB), Christian-Albrechts-University of Kiel, Kiel, 24118 Germany; 59 Bioinformatics Program, MGH Cancer Center and Department of Pathology, Boston, MA 02129 USA; 60 Cancer Genome Computational Analysis, Broad Institute, Cambridge, MA 02142 USA; 61 Endocrinology and Metabolism Department, Hadassah-Hebrew University Medical Center, Jerusalem, 91120 Israel; 62 Department of Psychiatry and Behavioral Sciences, SUNY Upstate Medical University, Oneida, NY 13421 USA; 63 Institute for Genomic Medicine, Columbia University Medical Center, Hammer Health Sciences, 1408, 701 West 168th Street, New York, NY 10032 USA; 64 Department of Genetics & Development, Columbia University Medical Center, Hammer Health Sciences, 1602, 701 West 168th Street, New York, NY 10032 USA; 65 Centro de Investigacion en Salud Poblacional. Instituto Nacional de Salud Publica MEXICO, Mexico, 62100 Mexico; 66 Lund University, Lund, SE-221 00 Sweden; 67 Institute for Molecular Medicine Finland (FIMM), HiLIFE, University of Helsinki, Helsinki, 00014 Finland; 68 Lund University Diabetes Centre, Lund, SE-214 28 Sweden; 69 Human Genetics Center, University of Texas Health Science Center at Houston, Houston, TX 77030 USA; 70 Department of Neurology, Columbia University, New York, NY 10032 USA; 71 Institute of Biomedicine, University of Eastern Finland, Kuopio, 70210 Finland; 72 Department of Psychiatry, PL 320, Helsinki University Central Hospital, Lapinlahdentie, 00 180 Helsinki, Finland; 73 Department of Medical Epidemiology and Biostatistics, Karolinska Institutet, Stockholm, 171 77 Sweden; 74 Icahn School of Medicine at Mount Sinai, New York, NY 10029 USA; 75 Department of Neurology, Helsinki University Central Hospital, Helsinki, 00290 Finland; 76 Department of Public Health, Faculty of Medicine, University of Helsinki, Helsinki, 00014 Finland; 77 Center for Genome Science, Korea National Institute of Health, Chungcheongbuk-do, 363-951 Republic of Korea; 78 MRC Centre for Neuropsychiatric Genetics & Genomics, Cardiff University School of Medicine, Hadyn Ellis Building, Maindy Road, Cardiff, CF24 4HQ UK; 79 National Heart and Lung Institute, Cardiovascular Sciences, Hammersmith Campus, Imperial College London, London, SW3 6LY UK; 80 Department of Health, THL-National Institute for Health and Welfare, 00271 Helsinki, Finland; 81 Section of Cardiovascular Medicine, Department of Internal Medicine, Yale School of Medicine, New Haven, CT 06510 USA; 82 Center for Outcomes Research and Evaluation, Yale-New Haven Hospital, New Haven, CT 06510 USA; 83 Division of Pediatric Gastroenterology, Emory University School of Medicine, Atlanta, GA 30322 USA; 84 Department of Internal Medicine, Seoul National University Hospital, Seoul, 03080 Republic of Korea; 85 Institute of Clinical Medicine, The University of Eastern Finland, Kuopio, 70210 Finland; 86 Kuopio University Hospital, Kuopio, 70210 Finland; 87 Department of Clinical Chemistry, Fimlab Laboratories and Finnish Cardiovascular Research Center-Tampere, Faculty of Medicine and Health Technology, Tampere University, Tampere, 33720 Finland; 88 The Mindich Child Health and Development Institute, Icahn School of Medicine at Mount Sinai, New York, NY 10029 USA; 89 Li Ka Shing Institute of Health Sciences, The Chinese University of Hong Kong, Hong Kong, China; 90 Hong Kong Institute of Diabetes and Obesity, The Chinese University of Hong Kong, Hong Kong, China; 91 Cardiovascular Research REGICOR Group, Hospital del Mar Medical Research Institute (IMIM), Barcelona, 08003 Catalonia Spain; 92 Department of Genetics, Harvard Medical School, Boston, MA 02115 USA; 93 Oxford Centre for Diabetes, Endocrinology and Metabolism, University of Oxford, Churchill Hospital, Old Road, Headington, Oxford, OX3 7LJ UK; 94 Wellcome Centre for Human Genetics, University of Oxford, Roosevelt Drive, Oxford, OX3 7BN UK; 95 Oxford NIHR Biomedical Research Centre, Oxford University Hospitals NHS Foundation Trust, John Radcliffe Hospital, Oxford, OX3 9DU UK; 96 F Widjaja Foundation Inflammatory Bowel and Immunobiology Research Institute, Cedars-Sinai Medical Center, Los Angeles, CA 90048 USA; 97 Atherogenomics Laboratory, University of Ottawa Heart Institute, Ottawa, ON K1Y 4W7 Canada; 98 Division of General Internal Medicine, Massachusetts General Hospital, Boston, MA 02114 USA; 99 Department of Clinical Sciences, University Hospital Malmo Clinical Research Center, Lund University, Malmo, 205 02 Sweden; 100 Lund University, Department of Clinical Sciences, Skane University Hospital, Malmo, 222 42 Sweden; 101 Instituto Nacional de Medicina Genómica (INMEGEN), Mexico City, 14610 Mexico; 102 Medical Research Institute, Ninewells Hospital and Medical School, University of Dundee, Dundee, DD1 9SY UK; 103 Department of Molecular Medicine and Biopharmaceutical Sciences, Graduate School of Convergence Science and Technology, Seoul National University, Seoul, 08826 Republic of Korea; 104 Department of Psychiatry, Keck School of Medicine at the University of Southern California, Los Angeles, CA 90033 USA; 105 Department of Psychiatry and Behavioral Sciences, Johns Hopkins University School of Medicine, Baltimore, MD 21205 USA; 106 Division of Genetics and Epidemiology, Institute of Cancer Research, London, SM2 5NG UK; 107 Medical Research Center, Oulu University Hospital, Oulu, Finland and Research Unit of Clinical Neuroscience, Neurology, University of Oulu, Oulu, 90014 Finland; 108 Research Center, Montreal Heart Institute, Montreal, Quebec, H1T 1C8 Canada; 109 Department of Medicine, Faculty of Medicine, Université de Montréal, Québec, H3T 1J4 Canada; 110 Department of Biomedical Informatics, Vanderbilt University Medical Center, Nashville, TN 37212 USA; 111 Department of Medicine, Vanderbilt University Medical Center, Nashville, TN 37212 USA; 112 Department of Biostatistics and Epidemiology, Perelman School of Medicine at the University of Pennsylvania, Philadelphia, PA 19104 USA; 113 Department of Medicine, Perelman School of Medicine at the University of Pennsylvania, Philadelphia, PA 19104 USA; 114 Center for Non-Communicable Diseases, Karachi, 75300 Pakistan; 115 National Institute for Health and Welfare, Helsinki, 00271 Finland; 116 Deutsches Herzzentrum München, München, 80636 Germany; 117 Technische Universität München, München, 80333 Germany; 118 Division of Cardiovascular Medicine, Nashville VA Medical Center and Vanderbilt University, School of Medicine, Nashville, TN 37232-8802 USA; 119 Department of Psychiatry, Icahn School of Medicine at Mount Sinai, New York, NY 10029 USA; 120 Department of Genetics and Genomic Sciences, Icahn School of Medicine at Mount Sinai, New York, NY 10029 USA; 121 Institute for Genomics and Multiscale Biology, Icahn School of Medicine at Mount Sinai, New York, NY 10029 USA; 122 Institute of Clinical Medicine, Neurology, University of Eastern Finland, Kuopio, 80101 Finland; 123 Department of Twin Research and Genetic Epidemiology, King’s College London, London, WC2R 2LS UK; 124 Departments of Genetics and Psychiatry, University of North Carolina, Chapel Hill, NC 27599 USA; 125 Saw Swee Hock School of Public Health, National University of Singapore, National University Health System, Singapore, 117549 Singapore; 126 Department of Medicine, Yong Loo Lin School of Medicine, National University of Singapore, Singapore, Singapore; 127 Duke-NUS Graduate Medical School, Singapore, 169857 Singapore; 128 Life Sciences Institute, National University of Singapore, Singapore, 117456 Singapore; 129 Department of Statistics and Applied Probability, National University of Singapore, Singapore, 117546 Singapore; 130 Folkhälsan Institute of Genetics, Folkhälsan Research Center, Helsinki, 00250 Finland; 131 HUCH Abdominal Center, Helsinki University Hospital, Helsinki, 00100 Finland; 132 Center for Behavioral Genomics, Department of Psychiatry, University of California, San Diego, CA 92093 USA; 133 Institute of Genomic Medicine, University of California, San Diego, CA 92093 USA; 134 Juliet Keidan Institute of Pediatric Gastroenterology, Shaare Zedek Medical Center, The Hebrew University of Jerusalem, Jerusalem, 91905 Israel; 135 Instituto de Investigaciones Biomédicas UNAM, Mexico City, 04510 Mexico; 136 Instituto Nacional de Ciencias Médicas y Nutrición Salvador Zubirán Mexico City, Mexico City, 14080 Mexico; 137 Radcliffe Department of Medicine, University of Oxford, Oxford, OX3 9DU UK; 138 Department of Gastroenterology and Hepatology, University of Groningen and University Medical Center Groningen, Groningen, 9713 The Netherlands; 139 Department of Physiology and Biophysics, University of Mississippi Medical Center, Jackson, MS 39216 USA; 140 Program in Infectious Disease and Microbiome, Broad Institute of MIT and Harvard, Cambridge, MA 02142 USA; 141 Center for Computational and Integrative Biology, Massachusetts General Hospital, Boston, MA 02114 USA; 142 Department of Psychiatry & Human Behavior, University of California Irvine, Irvine, CA USA

**Keywords:** Genetic variation, Genomics, Haplotypes, Genetic variation

Correction to: *Nature Communications* 10.1038/s41467-019-12438-5, published online 27 May 2020.

The original version of this Article omitted from the Genome Aggregation Database consortium the member Marquis P. Vawter, from the Department of Psychiatry & Human Behavior, University of California Irvine, Irvine, CA, USA. Additionally, the following was added to the Author Contributions: ‘All authors listed under The Genome Aggregation Database Consortium contributed to the generation of the primary data incorporated into the gnomAD resource’.

This has been corrected in both the PDF and HTML versions of the Article.

