# Supplementary material for: Landscape of multi-nucleotide variants in 125,748 human exomes and 15,708 genomes
Source: Nat Commun. 2020 May 27;11:2539. doi: 10.1038/s41467-019-12438-5 (PMC7253413; doi:10.1038/s41467-019-12438-5)
Supplement: Supplementary file 1 — Supplementary Information [file 41467_2019_12438_MOESM1_ESM.pdf]

“Landscape of multi-nucleotide variants in 125,748 human exomes and 15,708 genomes”

Wang et al., 2019

Supplementary information

Supplementary Methods (page 2 to 7)
Supplementary Tables 1~11 (page 8 to 14)
Supplementary Figures 1 ~ 21 (page 15 to 41)

Supplementary information for “Landscape of multi-nucleotide variants in 125,748 human exomes and 15,708 genomes”

Supplementary Methods:

MNV calling workflow

Using hail, we loaded the gnomAD data as a matrix table, pre-filtered the variants that are homozygote (hom) of reference allele (ref), applied the *window_by_locus* function to call MNVs, and used *aggregate* function to obtain the site level information. Our pipeline performs the equivalent calculation and outputs the same result as the python-like pseudo-code below, with orders of magnitude faster computational time compared to running a python script in local, achieved by cloud based parallel computing.

pseudo code:
let the window size be *k*, and a snv in position *i* be *snv_i*

```
for all position i in the genome:
    for j in [i+1, ... , k]:
        for every sample s carrying non-ref allele in both snv_i and snv_j:
        if (both snv_i and snv_j is hom):
            hom_snv_cnt_{s,i,j} = 1
        elif (only one of snv_i or snv_j is hom):
            het_snv_cnt_{s,i,j} = 1
        elif ((both snv_i or snv_j is het)
              & (snv_i and snv_j are phased in a same haplotype)):
            het_snv_cnt_{s,i,j} = 1
```

Allele counts for each site can be aggregated as

$$hom\_snv\_cnt_{i,j} = \sum_s hom\_snv\_cnt_{s,i,j}$$

$$het\_snv\_cnt_{i,j} = \sum_s het\_snv\_cnt_{s,i,j} .$$

For the genome data analysis, the number of MNV (or MNV count) in the main text denotes the total number of unique *(i, j)* pairs where $hom\_snp\_cnt_{i,j} + hom\_snp\_cnt_{i,j} > 0$ (i.e. the number of unique MNV sites). We took a slightly different approach to inferring MNV count for the exome analysis to focus on the functional consequence, by counting the number of MNV existing fully within a codon of a canonical transcript. We note that the number of unique MNV sites (31,197 sites) for exome coding regions is slightly lower than the MNV count (31,575 counts) we provide in the main text, as some MNVs affecting more than one canonical transcripts.

We set $k = 3$ for gnomAD exome and rare disease analysis, $k = 10$ for non-coding, and $k = 100$ for phase sensitivity analysis. The code above assumes no multiallelic variants for simplicity, but in reality we inspected multiallelic variants as well. This can be simple written by replacing *snv_i* with *snv_i_1*, *snv_i_2*, ... *snv_i_n* when there are n different snvs at position *i*, and re-writing the 4th line as

`*for all the SNV pairs (snv_i_*,snv_j_*)*` by letting "*" denote *1, 2, ... n.)*. This code assumes diploid status for the analyzed individual, but expanding to haploid status can be easily achieved by omitting the process of inspecting phase. We called MNVs in sex chromosomes for the exome and rare disease analysis. For the genome-wide analysis, we did not call the homozygotes whose phasing information is not assigned. We also saved the metadata such as the result of quality control (QC) for each site and sample, and chose to filter the variants or not depending on the study purpose.

Models and assumptions for calculating the proportion of MNV per biological mechanism

The fraction of non-SNV contribution for each MNV pattern, and its global probability were calculated as follows (note: in this section A or C is simply used according to the alphabetical order without any specification, and therefore does not mean adenine or cytosine):

1. We first took the number of 4 base pair contexts in the reference genome, as well as the median coverage of the region and defined them as follows:

$N(ABCD)$:= number of instances of the base pair context $ABCD$ in the reference genome

$c(x)$:= median coverage at position x

2. We know that the expected rate of detecting a variant is correlated to coverage at the site. We write this as $f(c(x))$.

For the calculation, we empirically approximated this relationship as $f(c) = tanh(c/25)$

3. Let the base line mutation rate defined by 3 bp context be $p_0(ABC \to ADC)$.Considering the coverage difference, the actual probability that we observe that change at the position x, where the 3 bp context of x is ABC is

$$p_x(ABC \to ADC) = p_0(ABC \to ADC) \cdot f(c(x)).$$

4. For a specific type of MNV $ABCD {-}{>} AEFD$ with B at position x, we calculate the relative probability of this happening as

$$p_x(ABCD \to AEFD) = p_x(ABC \to AEC) \cdot p_{x+1}(ECD \to EFD) + p_x(BCD \to BFD) \cdot p_{x+1}(ABF \to AEF)$$

If we assume the coverage of the bases next to each other is almost identical$f(c(x)) \approx f(c(x+1))$, then

$$p_x(ABCD \to AEFD) = \{p_0(ABC \to AEC) \cdot p_0(ECD \to EFD) + p_0(BCD \to BFD) \cdot p_0(ABF \to AEF)\} \cdot f^2(c(x))$$

5. The overall fraction of adjacent MNV of $ABCD {-}{>} AEFD$ can be calculated by integration over all the positions:

$$p(ABCD \to AEFD) = \sum_y p_y(ABCD \to AEFD),$$

where y are all the position across genome with B followed by C as the reference sequence.

6. The overall fraction of MNV of *BC* -> *EF*, where * can be arbitral of base pair (A, C, G, or T), can be calculated by summing over all the base patterns:

$$p(* BC * \to * EF *) := p(BC \to EF) = \sum_{a,d} p(aBCd \to aEFd)$$

7. In practice, if we write the average of $f^2(c)$for 4 bp pattern $ABCD$ as $cat(ABCD)$ (standing for “Coverage Adjustment Term”), the number of 4 bp count of ABCD in the reference genome as $N(aBCd)$), and the relative mutation probability for a single site as

$p_0(ABCD \to AEFD) = p_0(ABC \to AEC) \cdot p_0(ECD \to EFD) + p_0(BCD \to BFD) \cdot p_0(ABF \to AEF)$ then we can write the equation in 6. using summary statistics only, without actually scanning through the genome:

$$p(BC \to EF) = \sum_{a,d}\{p_0(aBCd \to aEFd) \cdot N(aBCd) \cdot cat(aBCd)\},$$

8. Now we have the probability matrix of 16x16 $:= P$, where each row denotes the reference and each column denotes the alternative alleles, and the entry corresponds to the relative probability of observing each type of MNV in the human genome. Let the observed MNV matrix be M (in practice, we let M be the number of MNVs that are not in repeat contexts), then we construct the “null matrix” N by multiplying the probability matrix with constant k:

$$N = k \cdot P$$

The approximation to estimate the parameter k will be discussed in 10. This null matrix, by definition, is the expected number of MNV for each MNV pattern, assuming single nucleotide substitution process, whose probability is defined by the 3 bp context, is the only driver of MNV. However, as seen in previous studies and our study, this overlooks some important MNV mechanisms such as polymerase zeta error and polymerase slippage at repeat junctions.

9. In order to include additional source of MNV such as polymerase zeta into the model, we added the “other contribution” term q_0, which depends only on the 2 bp context, and denote the new matrix as P'. Then the equation can be re-written as

$$p'_x(ABCD \to AEFD) = \{p_0(ABC \to AEC) \cdot p_0(ECD \to EFD) + p_0(BCD \to BFD) \cdot p_0(ABF \to AEF) + q_0(BC \to EF)\} \cdot f^2(c(x)).$$

Then following the same derivation step,

$$p'(BC \to EF) = \sum_{a,d}\{(p_0(aBCd \to aEFd) + q_0(BC \to EF)) \cdot N(aBCd) \cdot cat(aBCd)\}$$
$$= \sum_{a,d}\{p_0(aBCd \to aEFd) \cdot N(aBCd) \cdot cat(aBCd)\} + q_0(BC \to EF) \cdot N(BC) \cdot cat(BC)$$
$$= p(BC \to EF) + q_0(BC \to EF) \cdot N(BC) \cdot cat(BC)$$

10. Now we have the matrix P', and can let the null matrix be exactly equal to the observed matrix by letting $P' \cdot k = M$. However, this equation has $16 \cdot 9 + 1$ variables ($16 \cdot 9$ non zero element of matrix plus k), with only $16 \cdot 9$ constraints. Therefore, we manually set the parameter to make this calculation possible. Specifically, we hypothesize that “for the most common MNV pattern CA->TG, which we think is primarily driven by increased single base mutation rate by CpG methylation, there should be no significant extra factor contribution other than polymerase slippage at repeat junction, and therefore q_0 is approximately zero”. Specifically, we set $q_0(CA \to TG)$ to be 0, and calculated k and all the other q_0 terms:

$$k = \frac{m(CA \to TG)}{p(CA \to TG)},$$

$$q_0(BC \to EF) \cdot N(BC) \cdot cat(BC) = \frac{m(BC \to EF)}{k} - p(BC \to EF)$$

If we define the term $q_0(BC \to EF) \cdot N(BC) \cdot cat(BC)$ simply as $q(BC \to EF)$, and the matrix Q such that $Q_{i,j} = q_{i \to j}$, this can be directly used for the estimation of non spontaneous factor contribution.

Specifically, if we write $P' = P + Q$, then the fraction $\frac{Q_{i,j}}{(P_{i,j} + Q_{i,j})}$ directly provides the estimation of relative non-SNV contribution. Note that, for the calculations above, since the SNV mutation rates are typically in the order of 10^{-7} or lower, any higher order term was approximated as zero (i.e. we are assuming that there are no recurrent mutations).

11. In practice, mainly because of the fact that the mutation rate is not uniform across genomes even given the 3 bp context, the model produces some of the $q_0(i \rightarrow j)$ to be negative (i.e. we overestimate the underlying mutation rate by SNV combination) (Supplementary Fig 19). For such cases, since the fraction of overestimation is relatively small (less than 5% for most of the cases), we simply replaced the value with zero. Also, presumably because we set a manual threshold for the repeat context definition although in reality we know that the mutation rate increases as a function of repeat number (or sequence non-complexity in general), we might be underestimating the number of MNVs that originate from polymerase slippage at repeat contexts. Therefore, the fraction of "other" term should be interpreted carefully for the cases such as AA->TT, where both the fraction of repeat and others are high (i.e. the repeat count should rather be interpreted as the lower bound).

We also tried to incorporate additional complexity to the mutational model in two ways. First, we calculated the number of mutation sites in the gnomAD dataset per methylation status bin, and let the mutation rate differ as the function of methylation state (supplementary fig 20, (a) and (b)). Second, we expanded the sequence context model to 7 bp context rather than 3 bp, and calculated the mutation rate as a function of 7 bp context (supplementary fig 20, (c) and (d)). In both cases, the result was quite similar to the baseline case, suggesting that 3 bp context alone explains significant amount of MNV frequency.

In addition, we compared two models to adjust for coverage: the first assuming that the probability that each SNV of the MNV is detected is independent (therefore we adjust by the factor f^2), and the second assuming that the correlation between the detection of first SNV and the second is exactly equal to 1 (i.e. If we miss the first SNV by sequencing error due to low coverage, we also miss the second SNV that are next to it. Therefore we adjust by the factor f, without squaring). Although these two models differ in their assumptions, the difference was minor (>99% consistency in the final count).

Lastly, although theoretically the rate of MNV creation by SNV combination in a single generation is at most in the order of 10^{-15} ($10^{-8} \cdot 10^{-7}$in the case of non-CpG transition followed by a CpG transition), in practice we observe several orders of magnitude higher mutation rate overall. Our model takes relatively strong assumption that there is a "general" constant factor that pushes up the MNV mutation rate for all the MNV patterns. Further experiments to deepen our understanding of human genome mutation rate will be required to improve the assumptions made in our study.

Models and assumptions for estimation of the global MNV rate per substitution pattern

When assuming a fixed effective population size, no recurrent mutations, and the uniformity of the mutation rate across genome, the single base pair mutation rate $\mu_{SNV}$is known to be approximated as proportional to the number of the sites where mutations were observed (=$N(SNV)$).

$$\mu_{SNV} \propto N(SNV) = c_0 \cdot N(SNV)$$

In order to expand this basic model to MNV rate calculation globally and per MNV patterns, we expanded the model in two ways based on previous work (Kaplanis *et al*[2]) method.

1. Under the assumption that there is no recurrent mutation and therefore the allele frequency of constituent SNVs are equal if and only if they originate from an MNV event in a single generation, the global mutation rate of adjacent MNV can be written using $N(MNV)$ that denotes the number of 2 bp sites where one-step MNVs were observed:

$$\mu_{MNV} \propto N(MNV) \ = \ c_0 \cdot N(MNV)$$

(And the constant factor c_0 is same for two equations above, because of the fixed population size assumption and the uniform reference genome)

Since we know the SNV mutation rate from previous researches ($1.2 \cdot 10^{-8}$), we can simply calculate the global MNV mutation rate as

$$\mu_{MNV} \ = \ \mu_{SNV} \cdot \frac{N(MNV)}{N(SNV)},$$

Where $N(SNV)$ is the number of SNV sites.

In our data, this calculation resulted in the global MNV mutation rate of

$$\mu_{MNV} \ = \ 1.2 \cdot 10^{-8} \cdot \frac{488236}{199611214} \ = 2.94 \cdot 10^{-11}.$$

2. Under additional assumption that recurrent mutation is negligible for every 2 bp context (assuming that, although we know the single nucleotide mutation rate of CpG is different from other 2 bp context, the difference is minor compared to the MNV mutation rate.), we can compare the MNV mutation rate for different substitution patterns by letting the factor c_0 to be proportional to the inverse of the number of 2 bp count in the reference genome, rather than being a constant. We also account for slightly different coverage per 2 bp context, and write it as $cat(XY)$ (standing for "Coverage Adjustment Term", as in the previous section, but this time defined by 2 bp rather than 4 bp context). Then, by replacing the c_0 with $\frac{c}{cat(XY) \cdot N(XY)}$ (Following the intuition that, given the same number of observed MNVs, the mutation rate is higher if there are fewer reference 2 bp counts and/or lower coverage), and letting the mutation rate of XY->ZW given a sequence context XY per generation to be $\mu_{XY\to ZW}$, we can write the mutation rate as:

$$\mu_{\mathrm{XY\to ZW}} = \ c \cdot \frac{N(MNV_{XY\to ZW})}{cat(XY) \cdot N(XY)}$$

Here, the newly appeared constant term c is a scaling factor assumed to be uniform across different 2 bp context, and also when calculating the global MNV mutation rate. Specifically, if we use the term * to denote arbitral base pair (either A, C, G, or T), the global MNV mutation rate calculated in the 1 can be rewritten as:

$$\mu_{MNV} = 2.94 \cdot 10^{-11} = \ \mu_{**\to **} = c \cdot \frac{N(MNV_{**\to **})}{cat(**) \cdot N(**)},$$

Where $N(MNV_{**\to **})$ is identical to $N(MNV \ )$ in 1., $N(**)$, the number of 2 bp count in the reference genome, is equal to the number of reference genome length minus one, and the $cat(**)$ can be easily calculated from the median coverage of the entire genome.

With this, for all the 78 patterns of reference and alternative 2 bp, we can calculate the mutation rate as a function of $N(MNV_{XY\to ZW})$, $N(XY)$ and $cat(XY)$ (i.e. the number of MNV site of pattern XY->ZW, the number of 2 bp count of XY in the reference genome, and the mean of median coverage of XY), by deleting the constant term c from two equations above:

$$\mu_{XY\to ZW} = \ c \cdot \frac{N(MNV_{XY\to ZW})}{cat(XY) \cdot N(XY)} = \ 2.94 \cdot 10^{-11} \cdot \frac{cat(**) \cdot N(**)}{N(MNV_{**\to **})} \cdot \frac{N(MNV_{XY\to ZW})}{cat(XY) \cdot N(XY)}$$

Finally, comparison with the *de novo* MNV rate from trio exome sequencing data (Kaplanis et al.) was performed as follows. Kaplanis et al. estimated the global MNV mutation rate for the MNV of distance 1 to 20 bp to be $1.78 \cdot 10^{-10}$per base pair per generation. In order to scale this to our unit, which is MNV mutation rate restricting to adjacent MNVs (MNV of distance 1 bp), per 2 bp per generation, we divided the number by 2, and further multiplied by the fraction of adjacent MNVs out of all the MNVs of distance 1 to 20 bp (both restricting to one-step MNV) they have discovered. Specifically, this resulted in $\frac{1.78 \cdot 10^{-10}}{2} \cdot \frac{6606}{(6606+1568+4802)} = 4.53 \cdot 10^{-11}$, where 6606, 1568 and 4802 are the number of MNVs of distance 1 bp, 2 bp, 3-20 bp they have discovered. This is $\frac{(4.53 \cdot 10^{-11})}{(2.94 \cdot 10^{-11})} = 1.54$ times higher than the estimation provided by our analysis. We assume one of the main reasons for this discrepancy is the fact that the model does not take recurrent mutations into account, and would thus miss some of the MNV events followed by a single base pair mutation event, which might be particularly prevalent when the MNV results in CpG creation. Also, different MNV calling methods, as well as filtering criteria, are likely to contribute to the difference. Since we have no phase information for ~15% of heterozygous SNV pairs from our genome sequencing data, we cannot rule out the possibility of underestimating the MNV mutation rate. As our sequencing technology as well as statistical and computational methods evolves, further analysis of this estimate would be valuable.

Analysis of distance d>2

In a similar way as we calculated the MNV density per genomic region, we calculated relative (overall) density of MNV against MNV of distance 10, for all the distance d=1..9 and all the MNV patterns. Specifically, the relative MNV density $D_r$of distance d, pattern $W(*)_{d-1}X \rightarrow Y(*)_{d-1}Z$, where $(*)_{d-1}$denotes any sequence of length $(d-1)$, is defined as:

$$D_r\,(W, X \rightarrow Y, Z \mid d) = \frac{N(W(*)_{d-1}X \rightarrow Y(*)_{d-1}Z)\;/\;N(W(*)_{d-1}X)}{N(W(*)_9X \rightarrow Y(*)_9Z)\;/\;N(W(*)_9X)} = \frac{N(W(*)_{d-1}X \rightarrow Y(*)_{d-1}Z)}{N(W(*)_9X \rightarrow Y(*)_9Z)} \cdot \frac{N(W(*)_9X)}{N(W(*)_{d-1}X)}$$

According to this definition, $D_r\,(W, X \rightarrow Y, Z \mid d) = k$ means that the probability of observing a mutation of $W(*)_{d-1}X \rightarrow Y(*)_{d-1}Z$ given a sequence context of $W(*)_{d-1}X$ is k times higher than the probability of observing a mutation of $W(*)_9X \rightarrow Y(*)_9Z$ given a sequence context of $W(*)_9X$. Also, the result of density calculation stratified by the functional annotation is available in Supplementary Fig. 21.
We did not expand our analysis to a range longer than 10 bp, because of the instability of read based phasing sensitivity and specificity (Supplementary Fig. 1). The base pattern in the supplementary figures denotes the reference and alternative 2 bp pattern, but does not specify the patterns of bases in between (Therefore, we do not exclude the possibility that an MNV of distance>2 is a subset of an MNV of larger window. For example, substitution of AAAA->TTTT would be counted as all of A,A->T,T of distance 1, 2, and 3).

Supplementary Tables

Supplementary Table 1. Summary of phasing sensitivity evaluation in percentage
The ones without bracket is for genomes (635 trios), and the ones with bracket is for exomes (5,785 trios)

Categ \ distance (bp)	1	2	..	10	..	100
% (both are phased)	87.9<br>*(87.8)*	86.6<br>*(85.1)*	..	82.8<br>*(84.3)*	..	9.44<br>*(19.4)*
%(has PBT)	63.6<br>*(58.0)*	57.7<br>*(53.6)*	..	55.2<br>*(51.5)*	..	26.1<br>*(16.2)*
%(agrees PBT)	99.8<br>*(99.9)*	99.9<br>*(99.9)*	..	99.8<br>*(99.8)*	..	54.6<br>*(94.8)*

Supplementary Table 2. Summary of phasing sensitivity evaluation, in raw number
The ones without bracket is for genomes (635 trios), and the ones with bracket is for exomes (5,785 trios)

Categ \ distance (bp)	1	2	..	10	..	20	..	100
all	14965204<br>(1208731)	8456418<br>(562814)	..	6291498<br>(416324)	..	5505414<br>(398460)	..	4577148<br>(211667)
Same PID	13039584<br>(1058230)	7110950<br>(484128)	..	5201962<br>(339647)	..	2431721<br>(125741)	..	105171<br>(878)
Has PBT	8199080<br>(733947)	4235837<br>(309744)	..	3000591<br>(212241)	..	2666366<br>(178192)	..	2281022<br>(104394)
MNV	12552120<br>(1008797)	6837388<br>(458031)	..	4931735<br>(321854)	..	2331338<br>(121872)	..	104207<br>(876)
MNV that has PBT	7251154<br>(638060)	3636804<br>(260447)	..	2508690<br>(174833)	..	1039791<br>(50561)	..	16932<br>(229)
MNV that agrees PBT	7243988<br>(636582)	3632590<br>(260149)	..	2505046<br>(174508)	..	1037416<br>(50406)	..	16048<br>(125)

Supplementary Table 3. The read depth statistics, for each of the phased and unphased pairs. Thresholds: site depth 10^5 for "low" and 10^6 for "high" for genome ($10^6$and$10^{7.5}$for exome). Odds ratio =3.20, Fisher's exact test $p < 10^{-100}$ for low, and odds ratio =2.33, Fisher's exact test $p < 10^{-100}$for high read depth in genome (2.19 and 1.51 for exome, $p < 10^{-100}$ and $p < 10^{-8}$). Chromosome 20 of randomly selected 10% of samples in gnomAD genome dataset, and all chromosome of 1% of samples in exome dataset was examined (we down sampled to roughly match the trio analysis in number while keeping the statistical power, and manually set the threshold by looking at the distribution).

Categ \ depth bin	low	middle	high
number in genome, for unphased pair (%)	796 (0.0308)	2159905 (83.7)	421327 (16.3)
number in genome, for phased pair (%)	1129 (0.00962)	10911850 (93.0)	821621 (7.00)
number in exome, for unphased pair (%)	292 (0.441)	58358 (88.1)	7564 (11.4)
number in exome, for phased pair (%)	1090 (0.290)	354753 (94.5)	19581 (5.22)

Supplementary Table 4. Description of the functional annotations used in the research
The interval length, percentage of the whole genome, mean (across sites) of the median coverage (across individuals) in gnomAD, the mean (across sites) methylation level of CpG sites, and the percentage of regions that falls in LCR, are annotated as separate columns.

Category	Interval length	% of genome	Coverage	Methylation level	% in LCR
TSS	55509841	0.019603	30.6	0.194	1.306
5’ UTR	19902177	0.007028	30.1	0.350	0.880
Promoter	94046133	0.033212	30.4	0.437	1.793
Enhancer	126128190	0.044541	31.1	0.448	1.407
TFBS	380345514	0.134316	31.2	0.502	1.520
H3K4me3	397134049	0.140244	31.3	0.532	1.664
DHS	492285933	0.173846	31.3	0.543	0.844
H3K9ac	376617506	0.132999	31.2	0.546	1.512
Coding	61730033	0.021799	31.2	0.584	0.721
H3K27ac	772035773	0.272638	30.9	0.664	2.002
H3K4me1	1242793221	0.438882	31.1	0.717	1.590
3’ UTR	40715655	0.014378	30.7	0.720	1.243
Intron	1123766231	0.396848	30.6	0.797	2.221
Transcribed	1022244971	0.360997	30.4	0.866	0.880

Supplementary Table 5. Summary of the study

RF stands for random forest filtering, LCR stands for low complexity region, and adj stands for adjusted threshold (GQ >= 20, DP >= 10, and have now added: allele balance > 0.2 for heterozygote genotypes) filtering for each sample. 129 variants in Rare disease exome had gained nonsense mutation or high CADD score and low gnomAD frequency, but none of them were likely to be causal after manual inspection.

Callset	Exome	Genome	Rare disease exome
Sample size	125,748	15,708	6,072
Filtering Criteria	RF / LCR / adj	RF / LCR / adj	N/A
Annotations	- amino acid change<br>- gene constraint	- sequence context<br>- methylation status<br>- functional category	- gene constraint<br>- CADD score<br>- frequency in gnomAD
Number of MNVs	31,575 (within codon)	1,792,248 (within 3 bp)	129 (Not likely causal)
Main usage	Functional impact analysis	Mutational mechanisms analysis	Clinical diagnosis

Supplementary Table 6. Summary of the numbers of different MNVs in this paper

<table>
<tr><td rowspan="4">Exome</td><td rowspan="3">within codon</td><td rowspan="2">pairs of 2 SNVs: 31,575</td><td>Changes codon interpretation: 18,756</td></tr>
<tr><td>Does not change: 12,819</td></tr>
<tr><td colspan="2">combination of 3 SNVs: 228</td></tr>
<tr><td>spanning 2 codons</td><td colspan="2">pairs of 2 SNVs:23,429</td></tr>
<tr><td rowspan="6">Genome</td><td rowspan="4">adjacent MNVs</td><td rowspan="4">1,223,006</td><td>one-step MNV: 488,236</td></tr>
<tr><td>In repetitive context: 38,504</td></tr>
<tr><td>Most common (CA->TG): 270,071</td></tr>
<tr><td>Least common (TA->GC): 988</td></tr>
<tr><td>distance < 3</td><td colspan="2">1,792,248</td></tr>
<tr><td>distance<11</td><td colspan="2">5,513,219</td></tr>
</table>

Supplementary Table 7. The number and the percentage of SNV pairs that are filtered out by LCR / adj filtering

Category	Subcategory	number of SNV pairs pre-LCR and adj filtering	number of SNV pairs in LCR / fails adj	% filtered because of in LCR / fails adj
Exome	within codon, 2SNVs	31,667	92	0.29
	within codon, 3SNVs	229	1	0.44
Genome, adjacent MNVs	all	1,287,642	64,636	5.02
	one-step MNVs	503,189	7,695	1.53
	In repetitive context	48,885	10,381	21.2
	Most common (CA->TG)	275,237	5,166	1.88
	Least common (TA->GC)	1,025	37	0.98
Genome, all MNVs of distance <11	all	6,261,326	748,107	12.0
	one-step MNVs	1,031,038	25,165	2.44
	In repetitive context	502,925	199,069	39.6

Supplementary Table 8. Table view of the validation of phasing accuracy of read-based phasing using trio-based phase information
We are showing the case where read based phase of SNV1 is 0|1, without loss of generality.

SNV1<br>SNV2<br>(read base phase)	SNV1<br>SNV2<br>(trio base phase)	consistent
0|1<br>0|1	0|1<br>0|1	True
0|1<br>0|1	0|1<br>1|0	False
0|1<br>0|1	1|0<br>0|1	False
0|1<br>0|1	1|0<br>1|0	True
0|1<br>1|0	0|1<br>0|1	False
0|1<br>1|0	0|1<br>1|0	True
0|1<br>1|0	1|0<br>0|1	True
0|1<br>1|0	1|0<br>1|0	False

Supplementary Table 9. Number of MNVs spanning across two codons
Comparison of numbers of pairs of SNVs falling within a single codon and spanning across two codons in gnomAD exome data is shown.

distance \ codon	within codon	spanning two codons	total
d=1	25,287	14,070	39,357
d=2	6,288	9,359	15,647
total	31,575	23,429	55,004

Supplementary Table 10. breakdown of functional consequence of SNV1 and SNV2, for the MNVs spanning across two codons

Note that two SNVs are in different codons. “Others” includes start lost, stop lost and stop retained variants.

SNV1 (codon1)	SNV2 (codon2)	number
synonymous	synonymous	709
synonymous	missense	7,464
synonymous	nonsense	213
missense	synonymous	7,789
missense	missense	6,442
missense	nonsense	243
nonsense	synonymous	234
nonsense	missense	255
nonsense	nonsense	15
		others: 65
		total: 23,429

Supplementary Table 11. Number of MNVs in each consequence category, for each population

categ\pop	nfe	amr	sas	fin	eas	afr	asj	oth	all
gained nonsense	203	69	86	20	38	65	18	35	407
gained missense	27	16	21	4	8	6	2	4	73
changed missense	6,969	3,199	3,214	950	2,100	2,599	835	1,587	14,103
partially changed missense	1,103	515	504	149	328	409	128	255	2,194
lost missense	80	29	39	8	25	29	11	19	156
Unchanged	6,408	3,078	3,113	879	1,913	2,519	689	1,445	12,819
Rescued nonsense	950	407	457	114	283	323	92	177	1,821
total	15,740	7,313	7,434	2,124	4,695	5,910	1,775	3,522	31,573*

*2 variants, one with gained stop loss, the other with rescued stop loss, are excluded in this table

Supplementary Figures:

a.

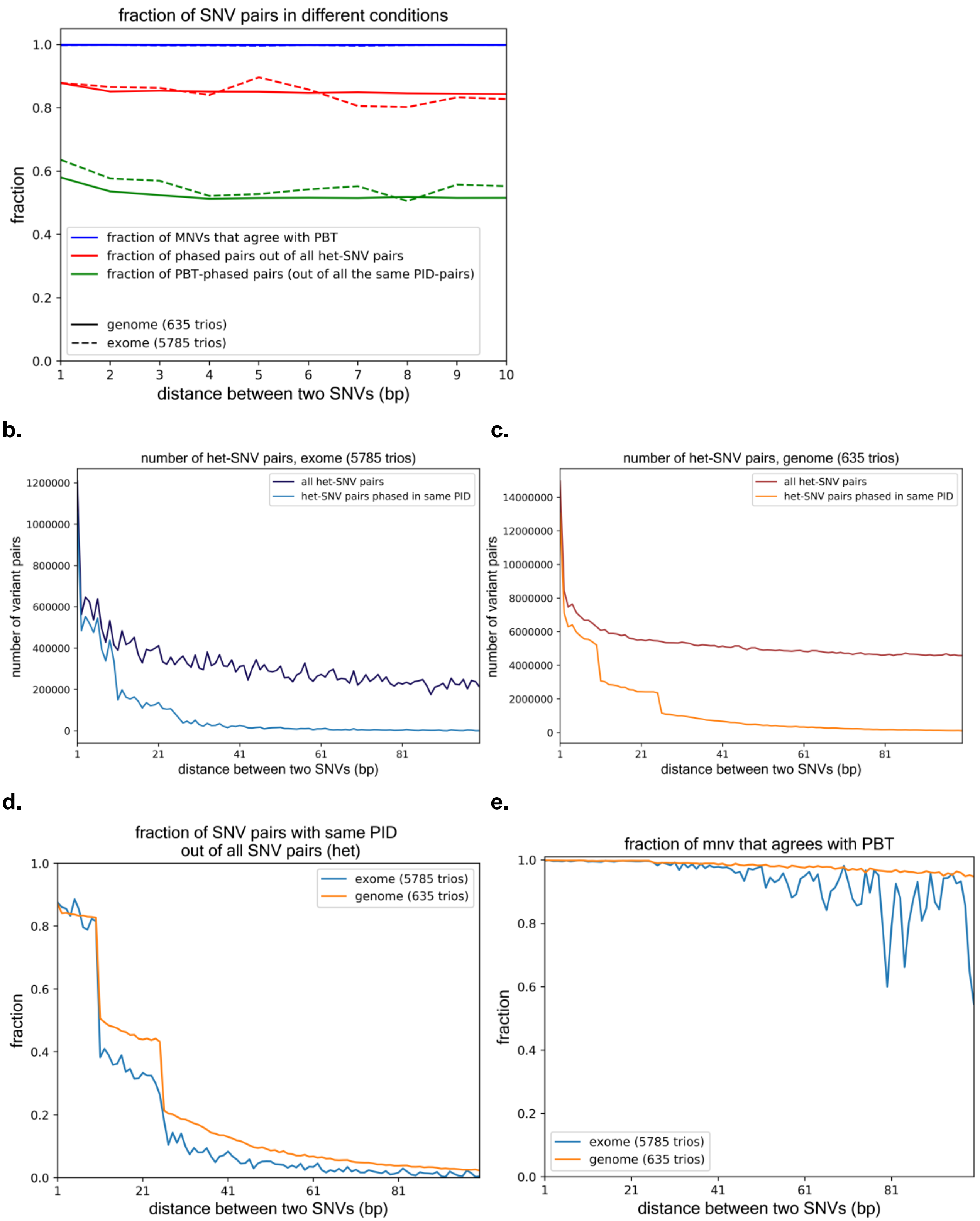

Supplementary Figure 1. Phasing quality as a function of distance

a, Fraction of MNVs that agrees with PBT (trio based phasing information), fraction of phased heterozygous SNV pairs that has phase information (by read base phasing), and fraction of trios that has trio based phasing information, up to 10 bp. Trio based phasing is by definition impossible when both of the parents are heterozygous, resulting in relatively low phasing sensitivity. **b-d,** The number **(b,c)** and the fraction **(d)** of heterozygous SNV pairs that has same Phase ID, out of all the heterozygous SNV pairs, up to 100 bp. The fraction significantly drops at >10 bp, reflecting limitations of read-based sequencing. **e,** Fraction of MNVs that agrees with trio based phase information, up to 100 bp.
For **(b)~(e)**, the estimation becomes unstable as the distance becomes large, because of both the limited number of sample size and phasing sensitivity.

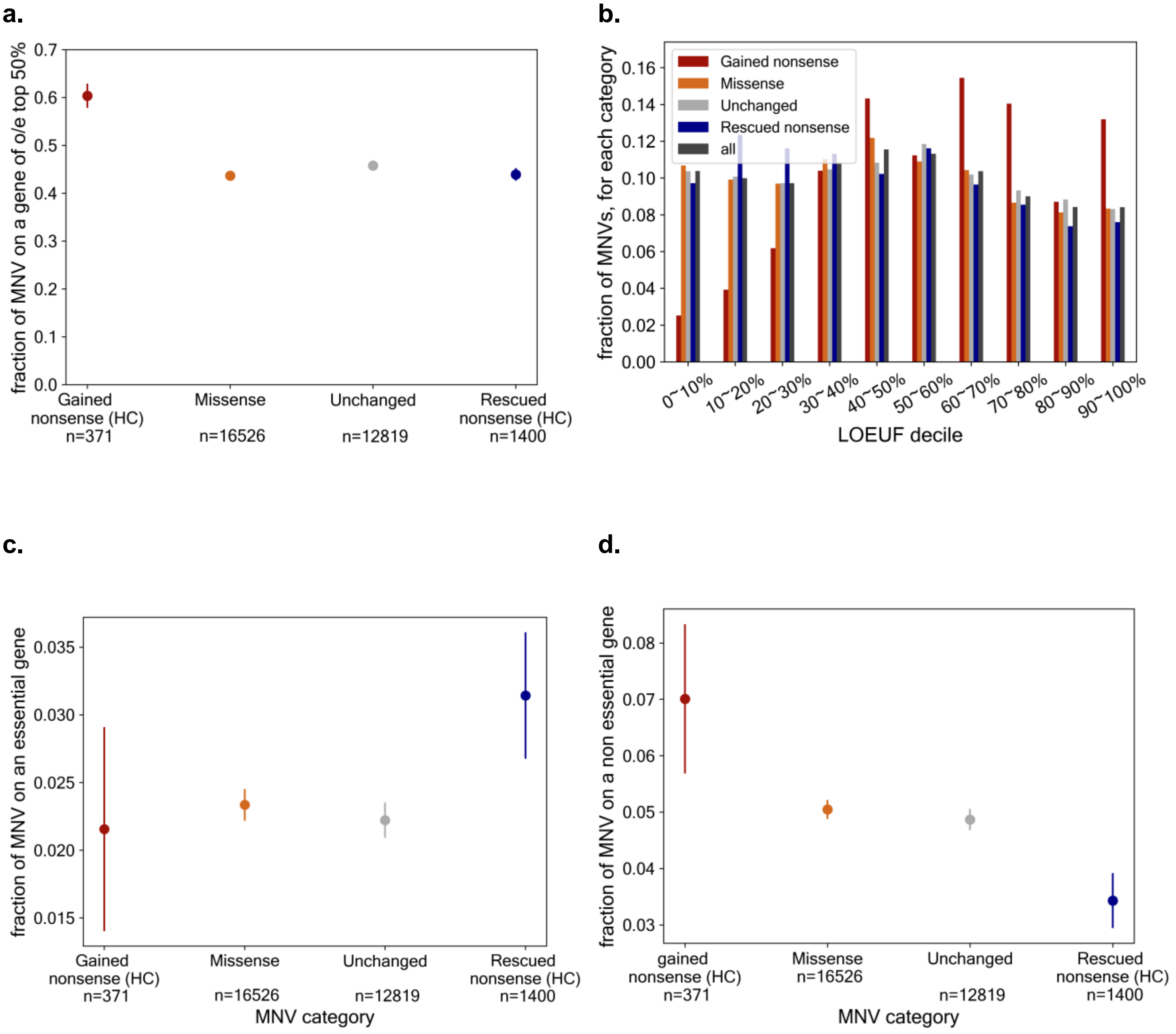

Supplementary Figure 2. MNV and gene constraint, cell essentiality

a, Fraction of MNVs that fall into un-constrained genes (n=9852) defined as top 50% in the observed vs expected number of loss-of-function in gnomAD data (LOEUF>0.926). The fraction for gained nonsense mutation is significantly higher than all the other classes combined (Fisher's exact test p-value = 7.04×10^{-5}). **b,** Fraction of MNVs of each functional category represented in each LOEUF decile. X axis shows the LEOUF decile, and y axis is fraction of MNVs that fall into genes of that specific LEOUF decile (adds up to 1 for each consequence category). **c,** Fraction of MNV that falls into essential genes (n=684), defined by knockout screening experiments using CRISPR/Cas[43,44]. The fraction for rescued nonsense mutation is significantly higher than all the other classes combined (Fisher's exact test p-value < 0.047). **d,** Fraction of MNV that falls into non essential genes (n=928), defined by knockout screening experiments using CRISPR/Cas[60,61] . The fraction for rescued nonsense mutation is significantly lower than all the other classes combined (Fisher's exact test p-value < 0.011).

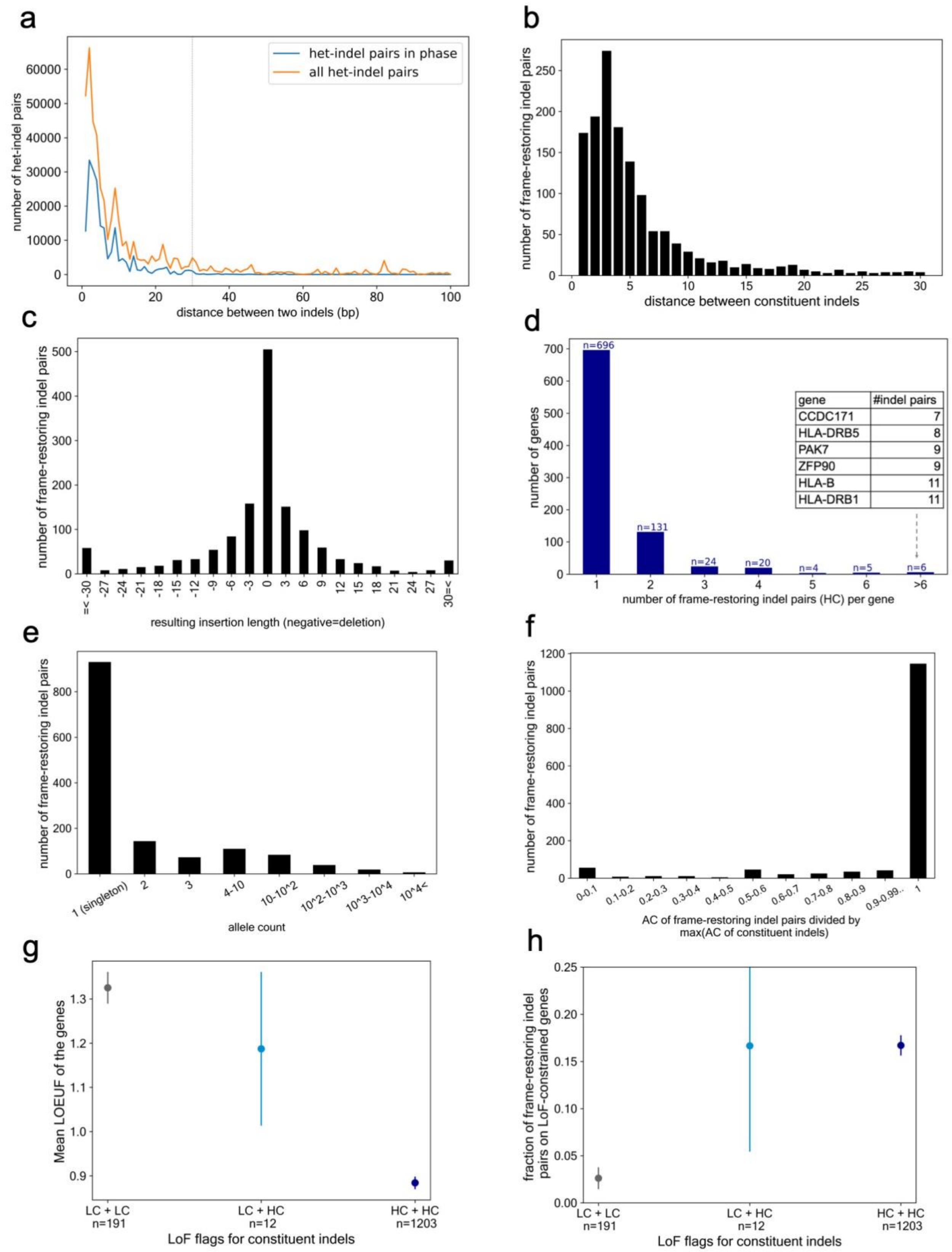

Supplementary Figure 3. Properties of frame-restoring indel pairs

a, The number of indel pairs (orange = all, blue = phased) is shown as a function of distance between the indels. We set the threshold distance to be 30bp as there are relatively few indel pairs past this distance. **b,** The distribution of the distance between indel pairs resulting in frame restoration (exome only, same for c~h). **c,** The distribution of the resulting insertion or deletion length for frame-restoring indel pairs. **d,** The number of frame-restoring indel pairs per gene, and the list of genes with more than six such variants. **e-f,** The allele count distribution of frame-restoring indels **(e)** and the distribution of allele counts divided by the maximum allele count of constituent SNVs **(f)**. The value is exactly 1 for 81.5% of overall frame-restoring indel pairs, suggesting that the majority of such indel events are likely the result of one-step mutational event. **g-h,** The mean LOEUF (constraint) score **(g)** and the fraction of LoF-constrained genes for frame-restoring indel pairs **(h)**, per combination of LOFTEE filters of the constituent indels.

a.

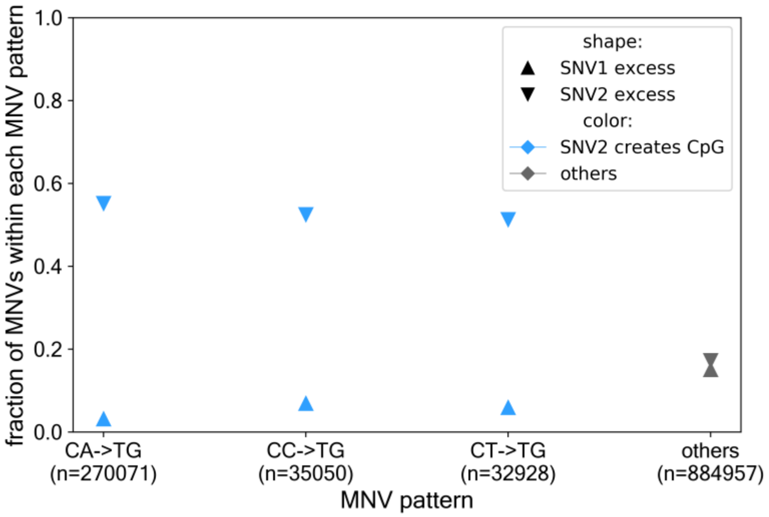

b.

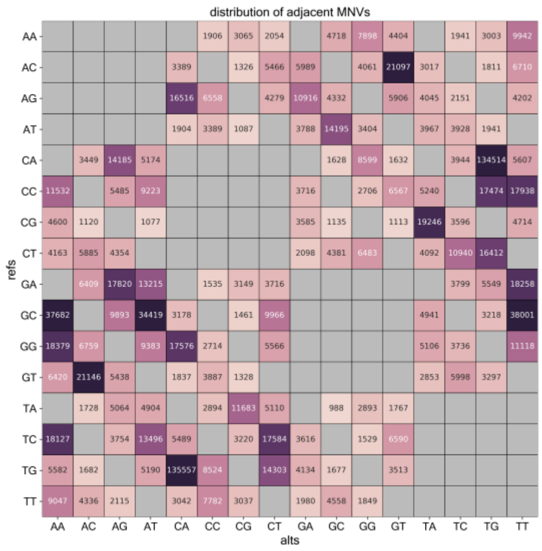

c.

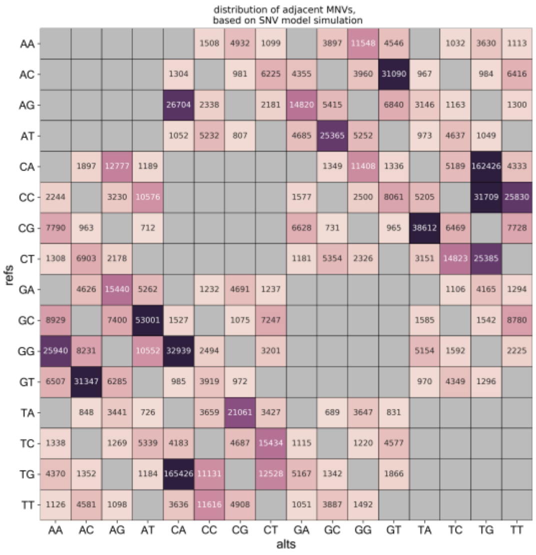

d.

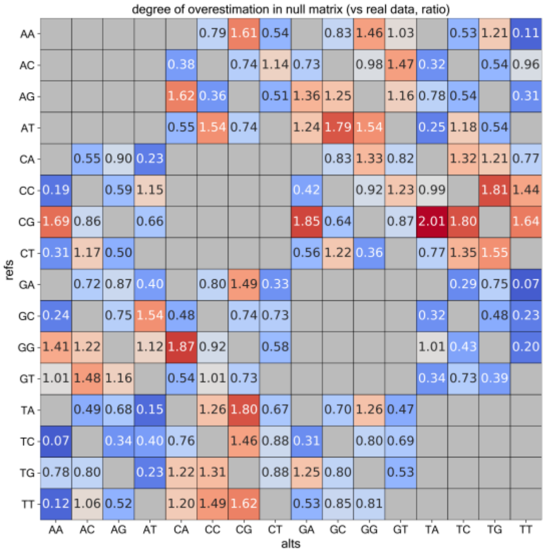

e.

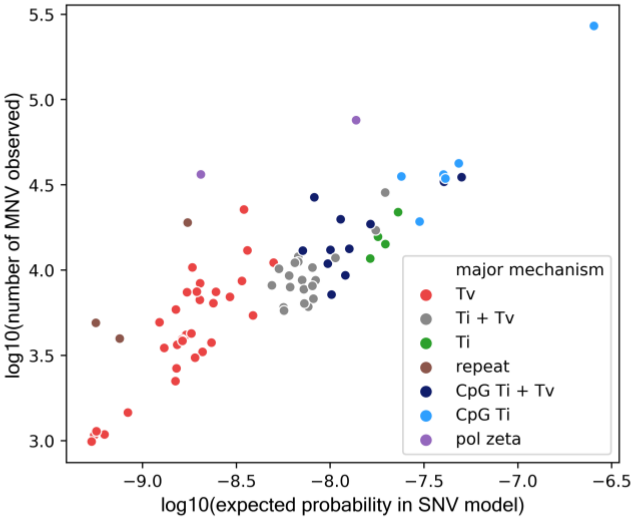

Supplementary Figure 4. Allele frequency difference of the SNVs in each MNV pattern, number of adjacent MNVs, and comparison with simulation by simple probabilistic model

a, Fraction of MNV in which allele count of only one of two constituent SNVs is larger than that of MNV (defined as "SNV excess"), indicating the non-uniform frequency of each step of two-step mutational processes. AC1 always corresponds to allele count of W->Y in WX->YZ. CT->TG in this figure is equivalent to AG->CA in other places (reverse complement was taken in this figure for the consistency with other MNV patterns that introduce CpG intermediate). **b,** Matrix representation of number of adjacent MNVs (reverse complements are not collapsed). Grey colored ones are by definition not MNV (SNV or not even a mutation). **c,** Matrix representation of number of adjacent MNVs (reverse complements are not collapsed), in a null model based on SNV mutation rate, assuming all MNV are consequence of two single nucleotide variation events. **d,** Enrichment compared to the null model based on SNV mutation rate. **e,** Scatter plot where x axis shows the relative probability of MNV based on null model (per generation), and y axis is the number of MNV observed in gnomAD data, both in log10 scale. The color represents the predicted major mechanism for each MNV pattern. Polymerase zeta and repetitive motifs are pushing up the number of MNV compared to the null model. Pearson correlation r=0.84 before removing polymerase zeta and repeat signature, and r=0.99 after removing those two signature (purple and brown), for linear regression in log space (black line).

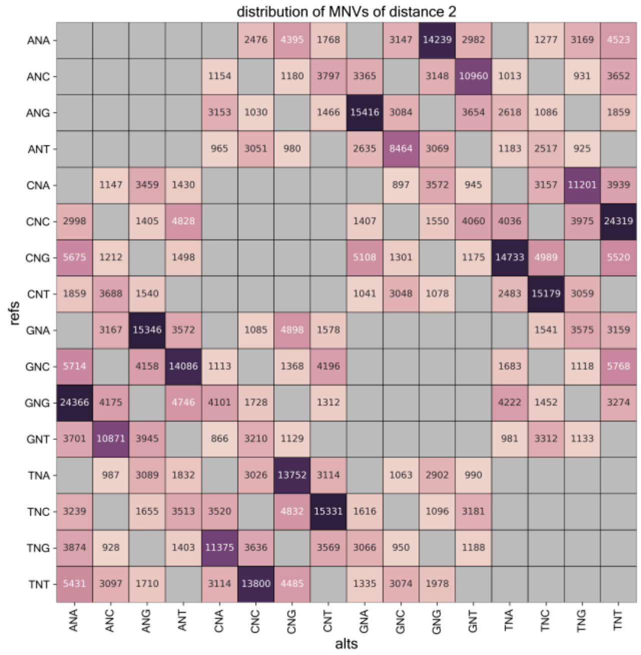
distribution of MNVs of distance 2
refs
alts
ANA
ANC
ANG
ANT
CNA
CNC
CNG
CNT
GNA
GNC
GNG
GNT
TNA
TNC
TNG
TNT

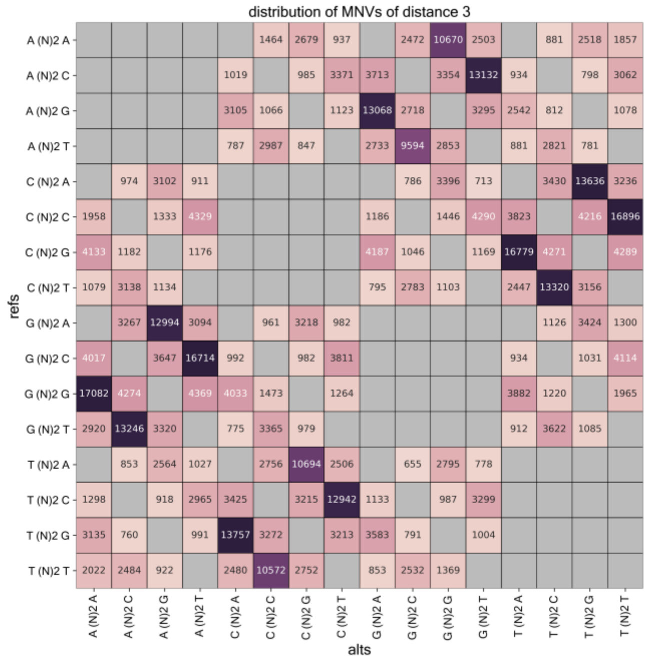
distribution of MNVs of distance 3
refs
alts
A (N)2 A
A (N)2 C
A (N)2 G
A (N)2 T
C (N)2 A
C (N)2 C
C (N)2 G
C (N)2 T
G (N)2 A
G (N)2 C
G (N)2 G
G (N)2 T
T (N)2 A
T (N)2 C
T (N)2 G
T (N)2 T

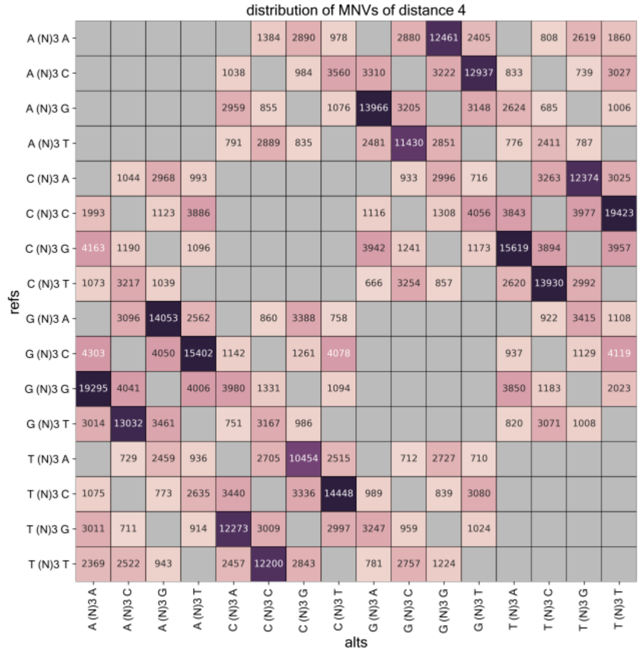
distribution of MNVs of distance 4
refs
alts
A (N)3 A
A (N)3 C
A (N)3 G
A (N)3 T
C (N)3 A
C (N)3 C
C (N)3 G
C (N)3 T
G (N)3 A
G (N)3 C
G (N)3 G
G (N)3 T
T (N)3 A
T (N)3 C
T (N)3 G
T (N)3 T

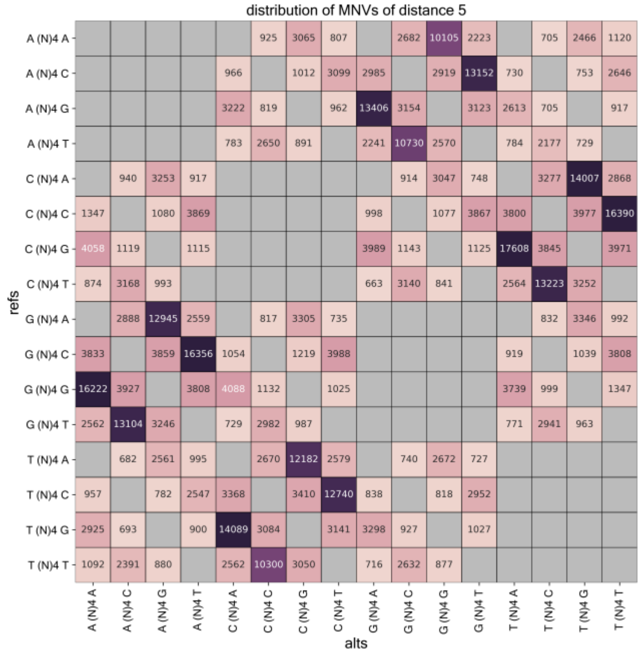
distribution of MNVs of distance 5
refs
alts
A (N)4 A
A (N)4 C
A (N)4 G
A (N)4 T
C (N)4 A
C (N)4 C
C (N)4 G
C (N)4 T
G (N)4 A
G (N)4 C
G (N)4 G
G (N)4 T
T (N)4 A
T (N)4 C
T (N)4 G
T (N)4 T

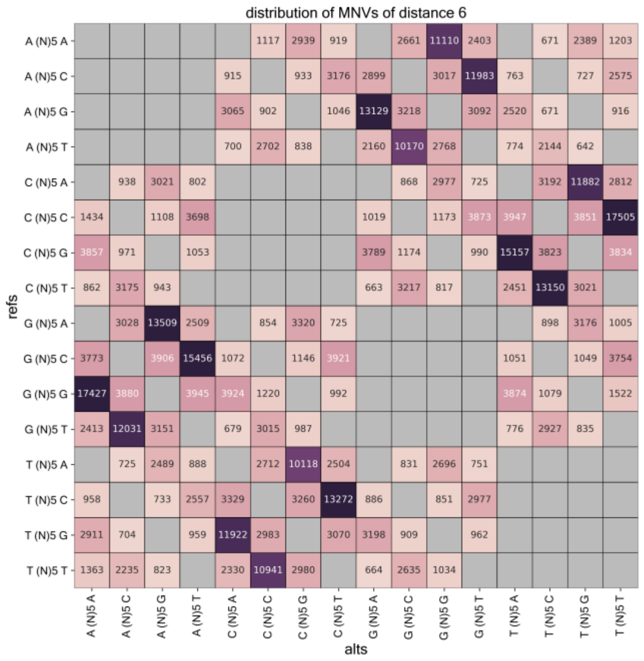
distribution of MNVs of distance 6
refs
alts

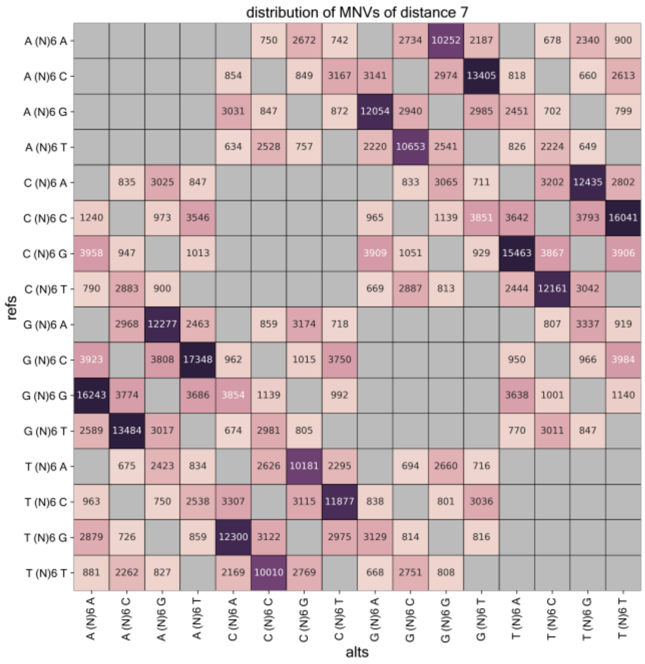
distribution of MNVs of distance 7
refs
alts

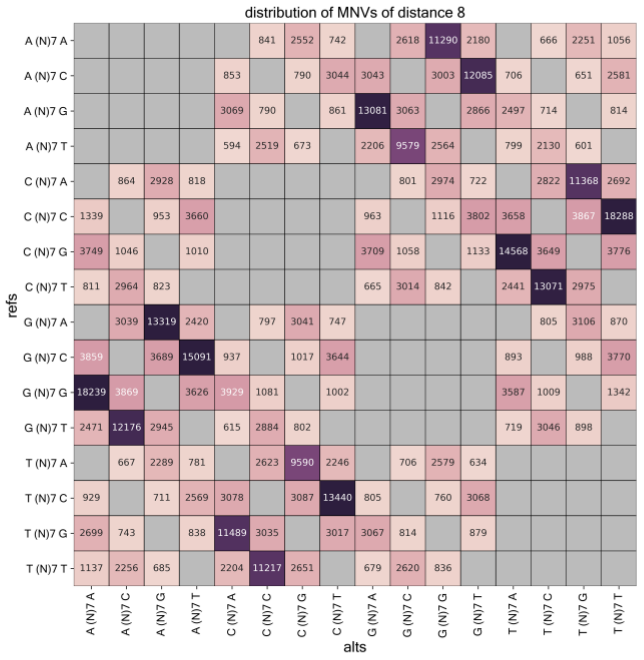
distribution of MNVs of distance 8
refs
alts

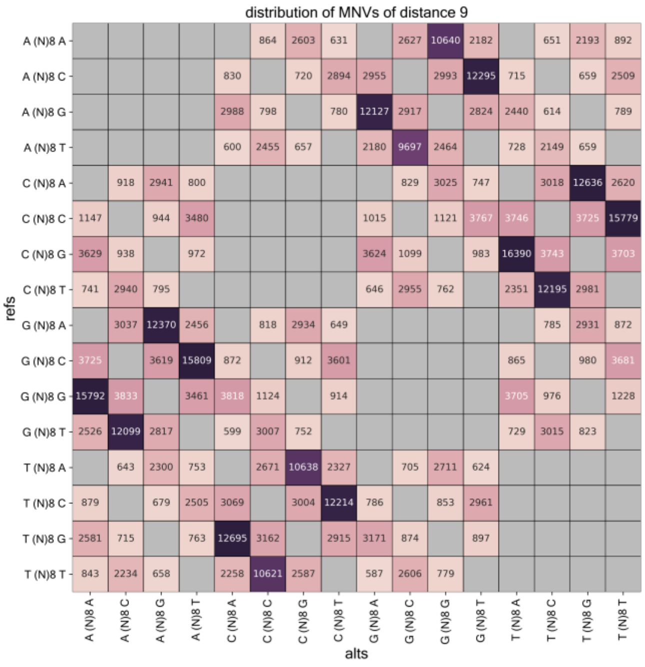
distribution of MNVs of distance 9
refs
alts

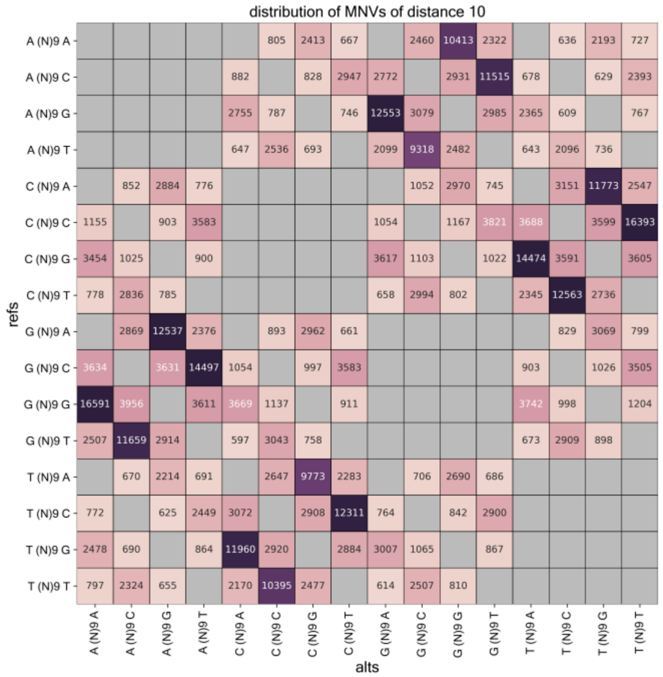

Supplementary Figure 5. Matrix representation of number of MNVs, up to 10 bp
Row denotes the reference, and the column denotes the alternative alleles. (N)M in the row and column means arbitral sequence of length M (e.g. (N)2 is one of {AA, AC, AG,..., TT}).

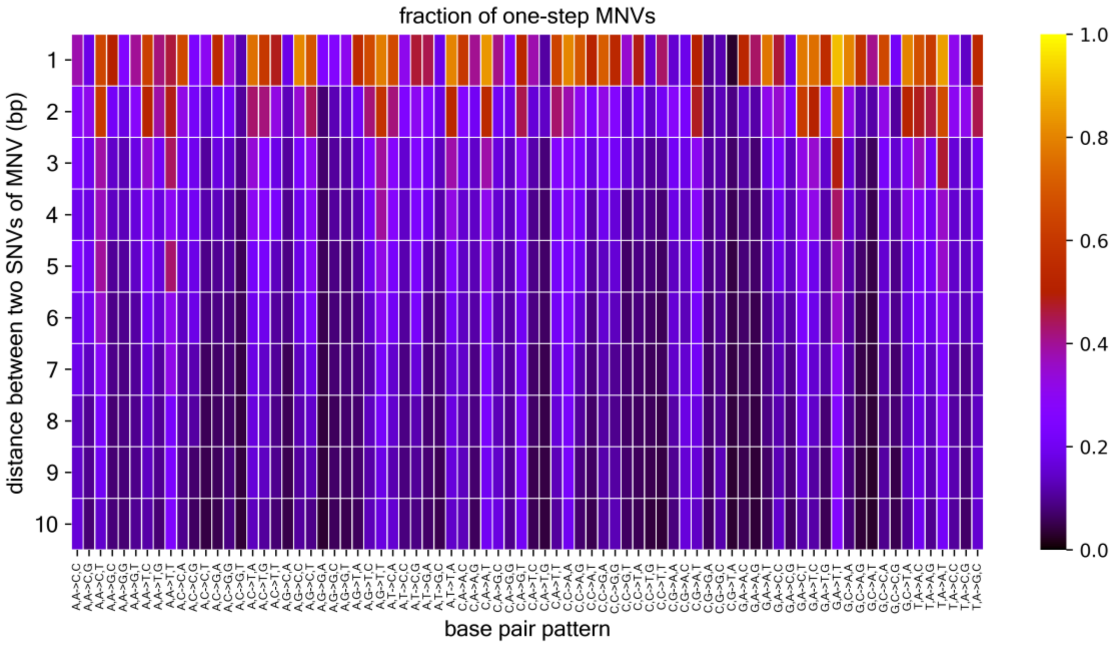

Supplementary Figure 6. Fraction of one-step MNV, up to 10 bp
Row denotes the distance between two SNVs of MNV, and column denotes the base pair pattern. The fraction is represented as color.

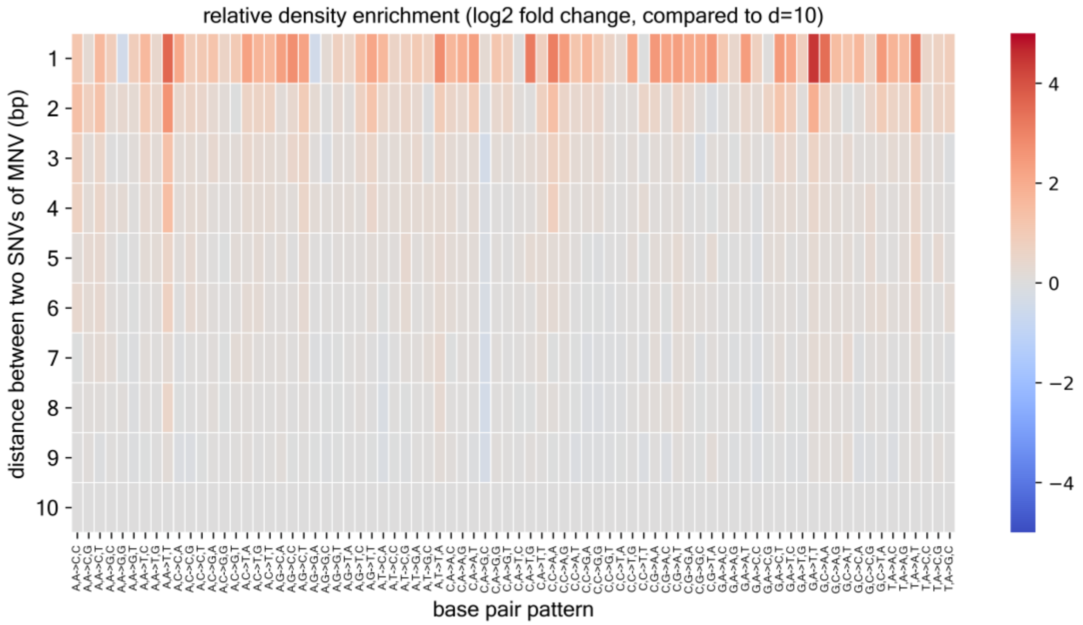

Supplementary Figure 7. Relative MNV density enrichment per MNV pattern, compared to 10 bp distance

Row denotes the distance between two SNVs of variant pairs, and column denotes the base pair pattern. The relative density enrichment is represented as color (color shifts in log scale). By definition for row=10, all the column entries are exactly equal to 1 (=0 in log scale).

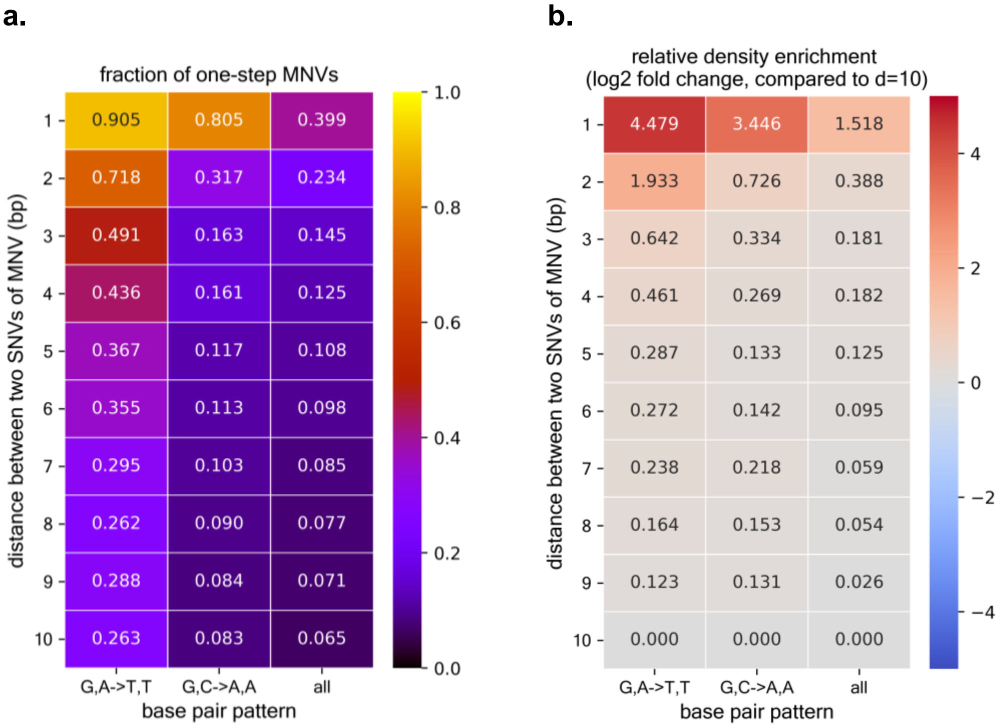

Supplementary Figure 8. Polymerase zeta signatures are observed for distance>1

Figure generated by taking two columns from **figure S6** and **S7**, and adding the overall level as comparison. The fraction of one-step MNV **(a)** and the density of MNV **(b)** for G,A->T,T and G,C->A,A, the MNV pattern known as polymerase zeta signature when the distance is 1 bp, are higher compared to the overall value, even for distance>1 (= not only GA->TT, but also GNA->TNT, GNNA->TNNT, … are enriched) (Fisher's exact test p-value < 0.05 up to distance 6 bp for the fraction of one-step MNV, and up to 9 bp for the relative density, compared to MNVs of same pattern, distance 10 bp). We do not fully exclude the possibility that the signals we observe are driven by sequence error or other artifact, since the fraction of variants that are filtered out tends to be high and the mean coverage is low for these variants, especially when two base pairs of MNV are not adjacent.

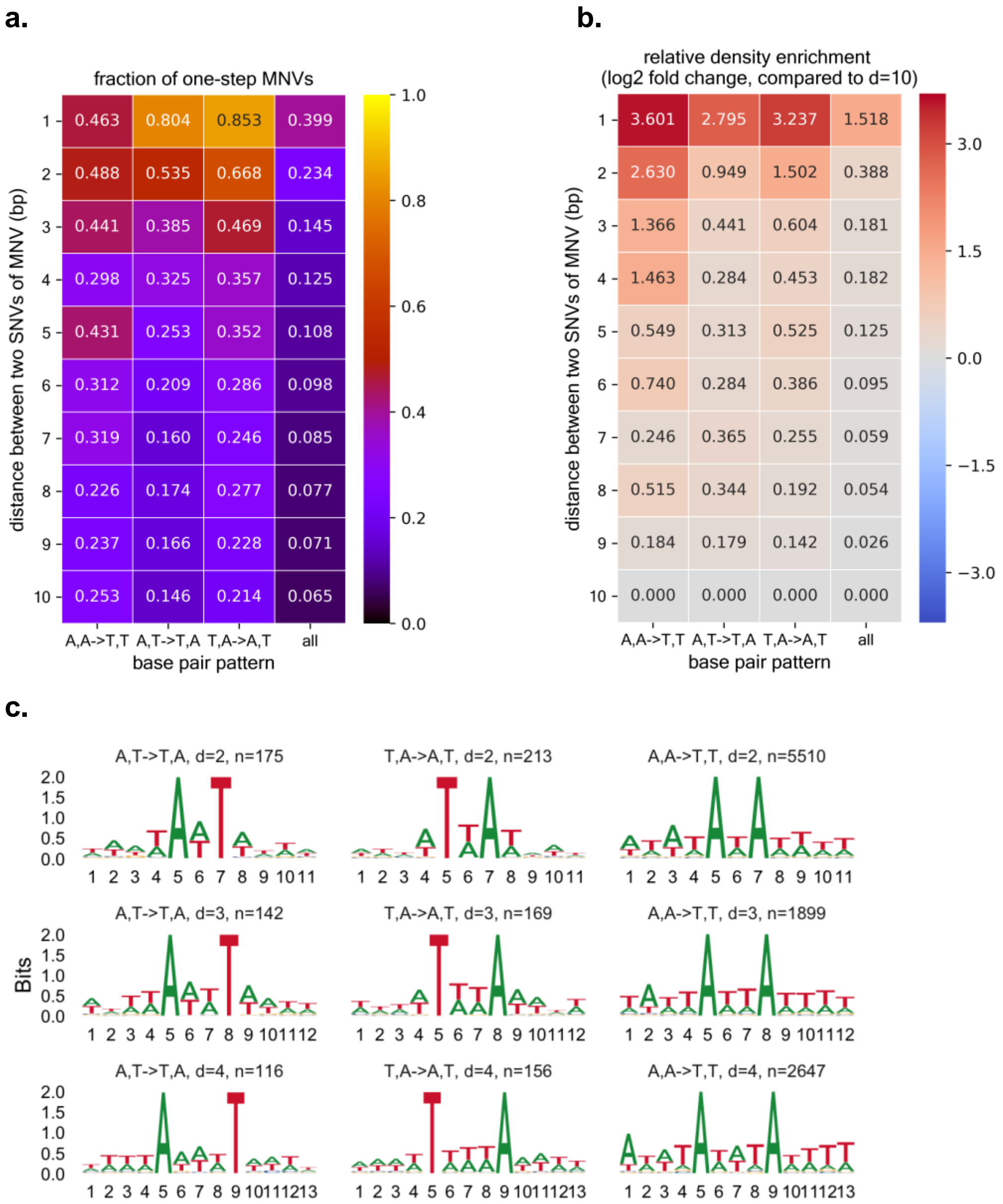

d.

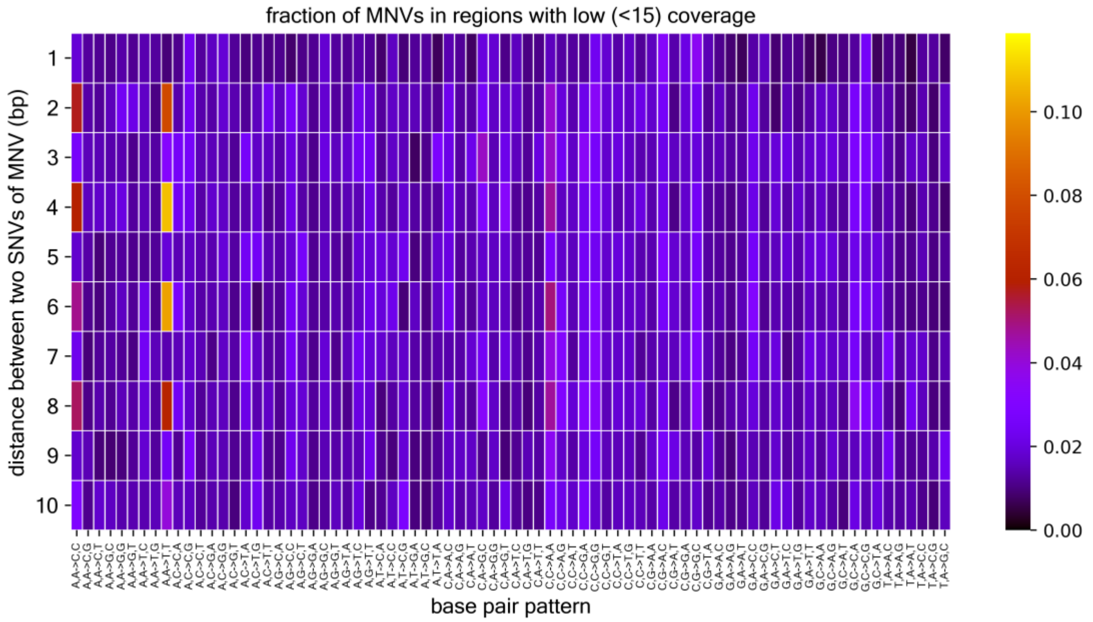

Supplementary Figure 9. Repeat signature are observed for distance>1, but some have low coverage

a, b, Figure generated by taking four columns from **figure S6** and **S7**, and adding the overall level as comparison. The fraction of one-step MNV **(a)** and the density of MNV **(b)** for the patterns classified as polymerase slippage signature when the distance is 1 bp, are higher compared to the overall value, even for distance>1 (Fisher's exact test p-value < 0.05 up to distance 3 bp for the fraction of one-step MNV, and up to 9 bp for the relative density, compared to MNVs of same pattern, distance 10 bp). We do not fully exclude the possibility that the signals we observe are driven by sequence error or other artifact, for same reason as **figure S8,** and as shown in **(d)**. **c,** bits logo of enriched repetitive sequence context in distance 2, 3 and 4 (x=relative base pair position). **d,** Fraction of MNV that are in a region of median coverage <15, up to 10 bp. Row denotes the distance between two SNVs of variant pairs, and column denotes the base pair pattern.

a. **b.**

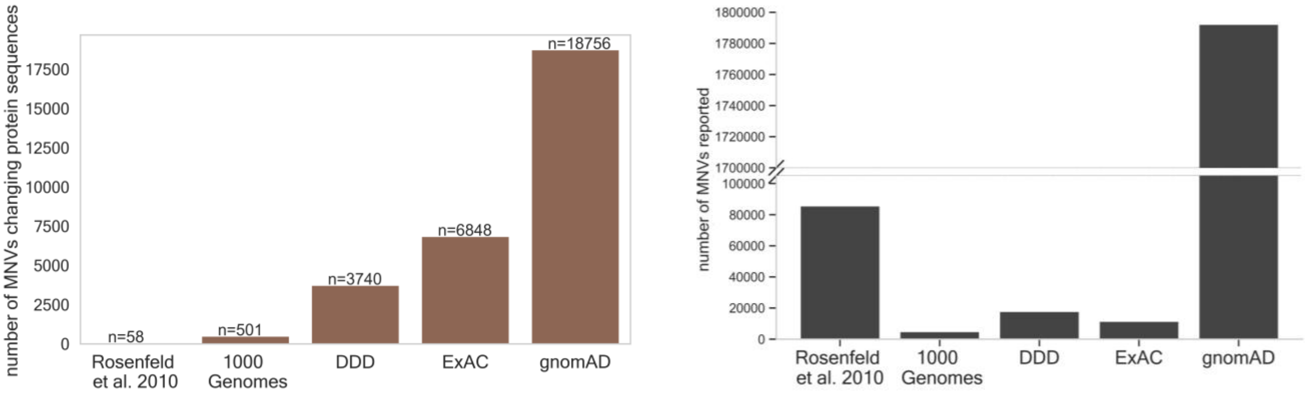

Supplementary Figure 10. Comparison of numbers of MNVs in different studies

Number of MNVs that change protein sequences **(a)** and the total number of MNVs (within 3 bp length) reported **(b)**, for five major studies to date: Rosenfeld et al. (2010)[3], 1000 Genomes[9,10], Deciphering Developmental Disorder (DDD)[2], Exome Aggregation Consortium (ExAC)[1], and genome Aggregation Database (gnomAD)[22]. For Rosenfeld et al. in **(a)**, we estimated the number from the total number of MNVs in exon, using the statistics from DDD and gnomAD. Note that **(b)** is not necessarily equal to the total number of MNVs discovered in the study, since some studies do not explicitly report non-coding MNVs. Also the statistics is subject to different variant calling and filtering criteria applied for different studies.

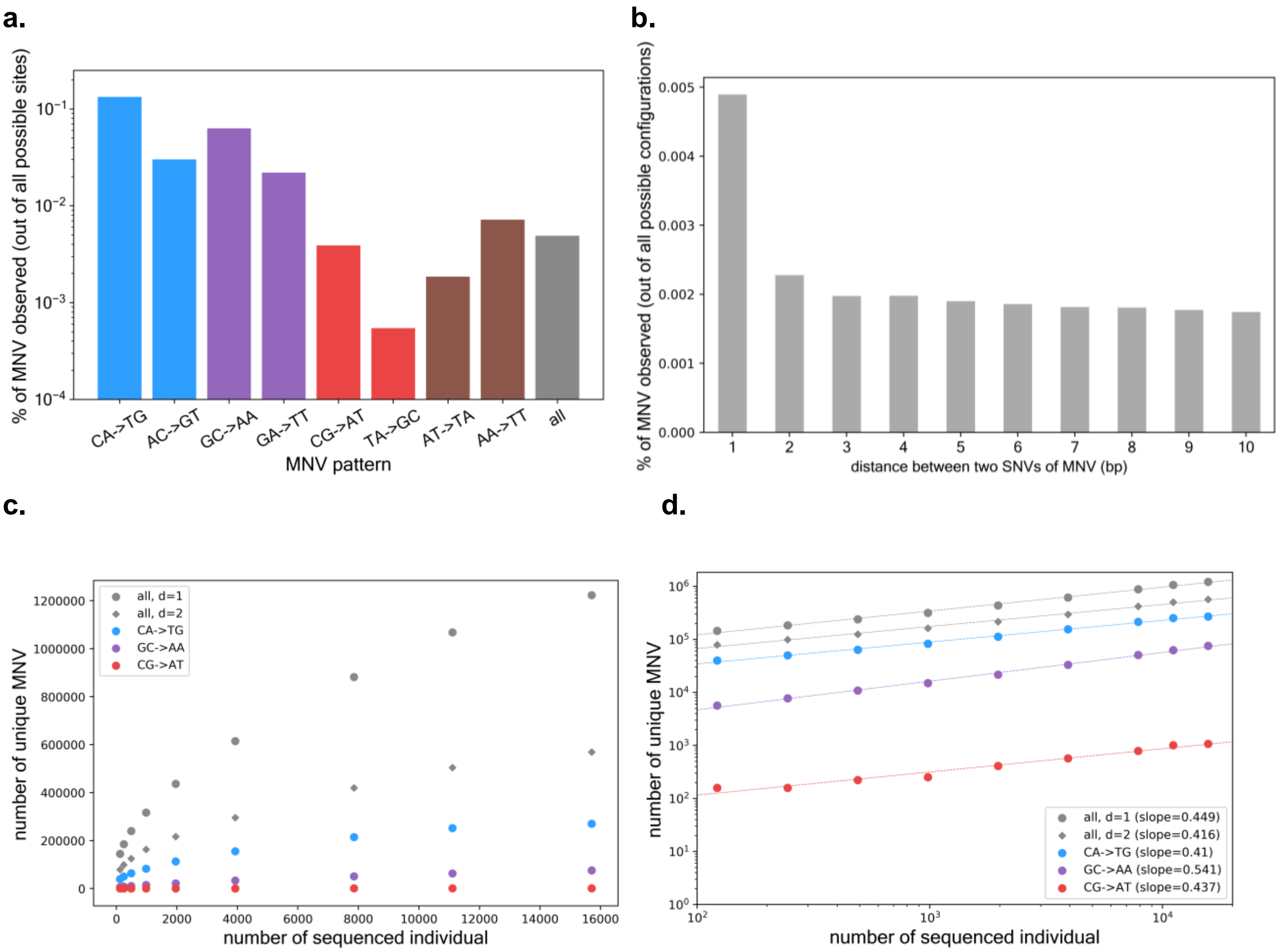

Supplementary Figure 11. percentage of MNV we have observed

a, Percentage of MNVs we have observed, per MNV pattern. Two patterns per major mutational mechanisms are shown as representations. Y axis is log scaled. **b,** Percentage of MNV we have observed, per distance. **c, d**, Number of MNV that are observed, as a function of down-sampled population size (median of 3 different random seeds. Error bars, denoting the maximum and minimum of 3 seeds, were constantly smaller than the dot size), in real **(c)** and log **(d)** scale. If we make a strong assumption that the log-linearity we observe in the current sample size holds true even with orders of magnitude higher sample size, we expect the percentage of all possible MNVs that we observe when we sequence the entire population (n=7.7×10^9) would be roughly 1.6%

a.

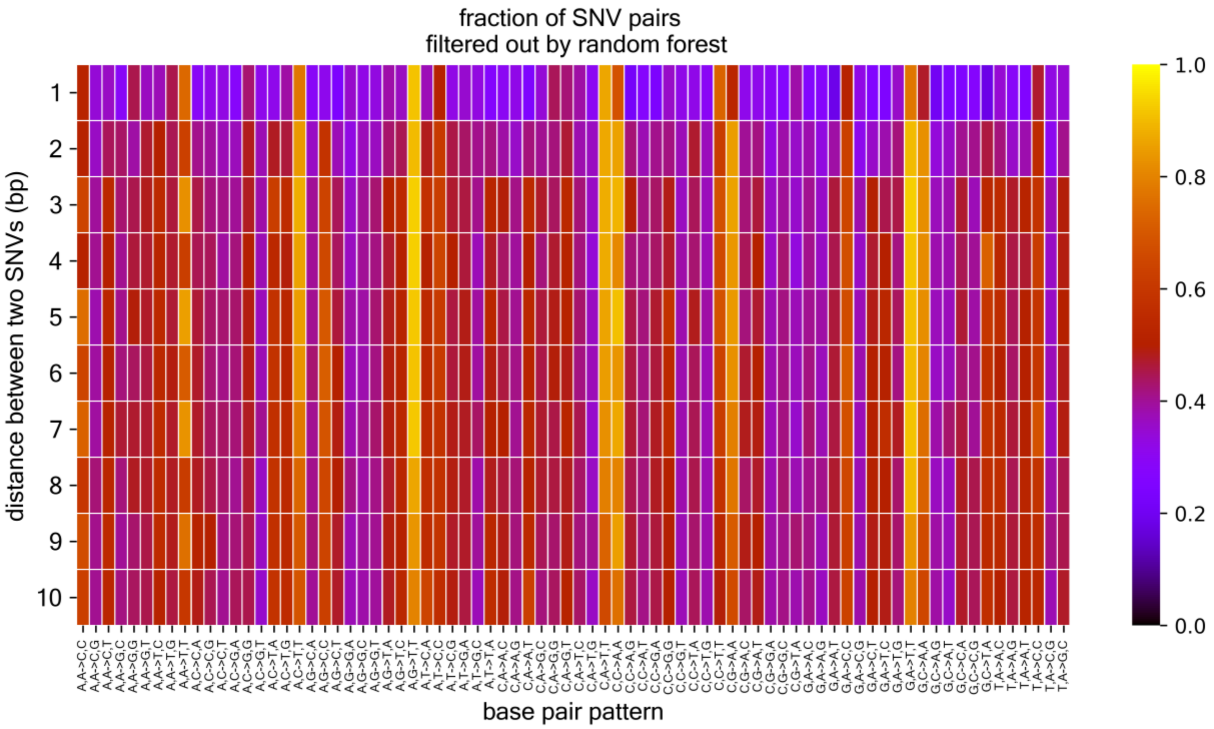
fraction of SNV pairs
filtered out by random forest
distance between two SNVs (bp)
base pair pattern
1.0
0.8
0.6
0.4
0.2
0.0

b.

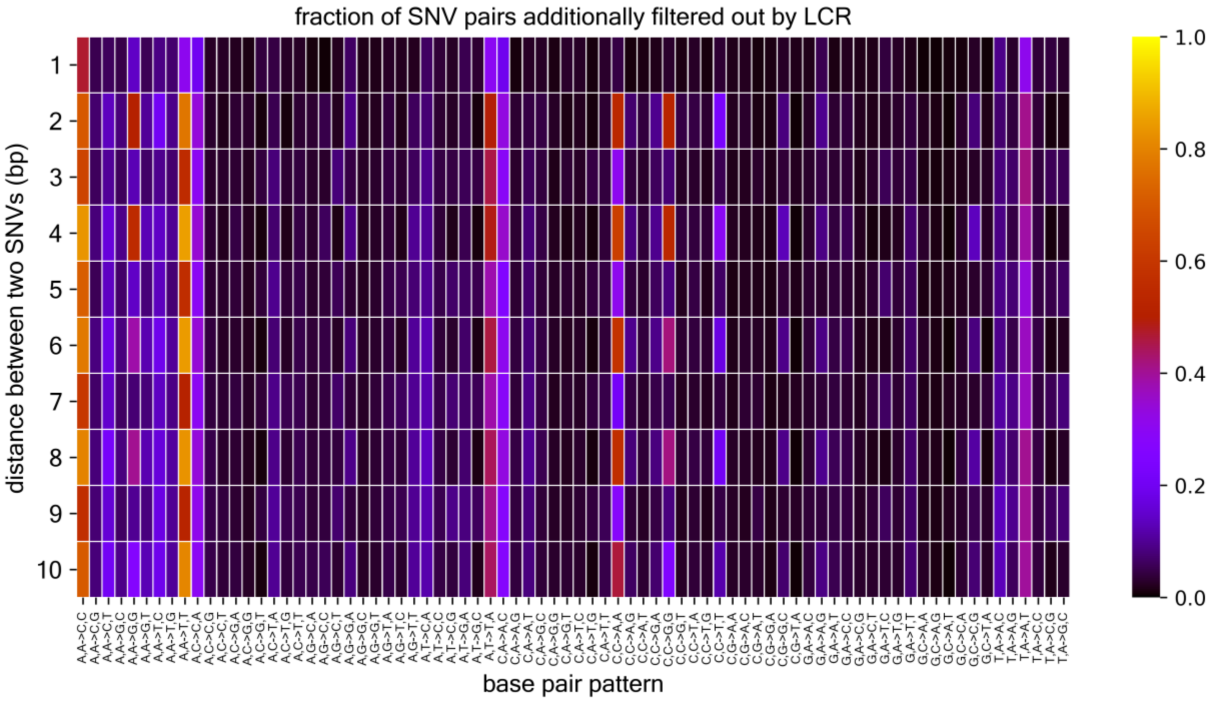
fraction of SNV pairs additionally filtered out by LCR
distance between two SNVs (bp)
base pair pattern
1.0
0.8
0.6
0.4
0.2
0.0

c.

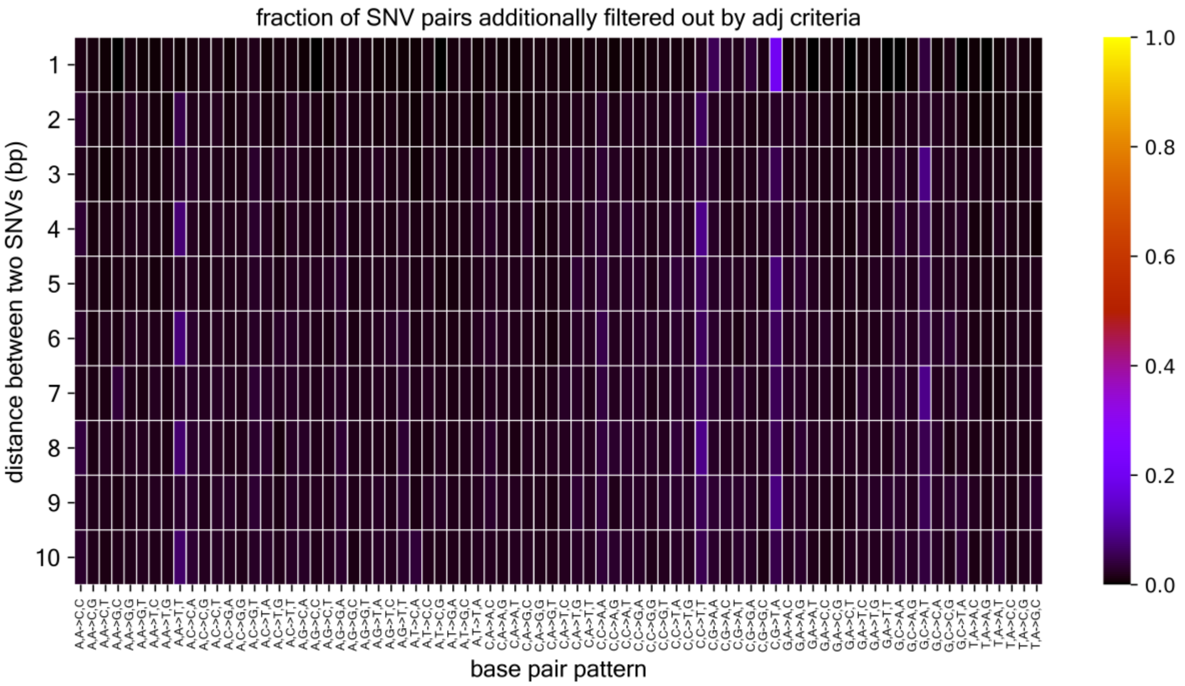

Supplementary Figure 12. Fraction of variant pairs that are filtered out by random forest (a) / by regional filter (LCR= low complexity region) (b) / by failing the adjusted criteria (c), up to 10 bp
Row denotes the distance between two SNVs of variant pairs, and column denotes the base pair pattern. The fraction is represented as color. The denominator is the number before filtering In **(a)**, the number after applying random forest filtering in **(b)**, and the number after applying random forest and LCR filtering in **(c)**.

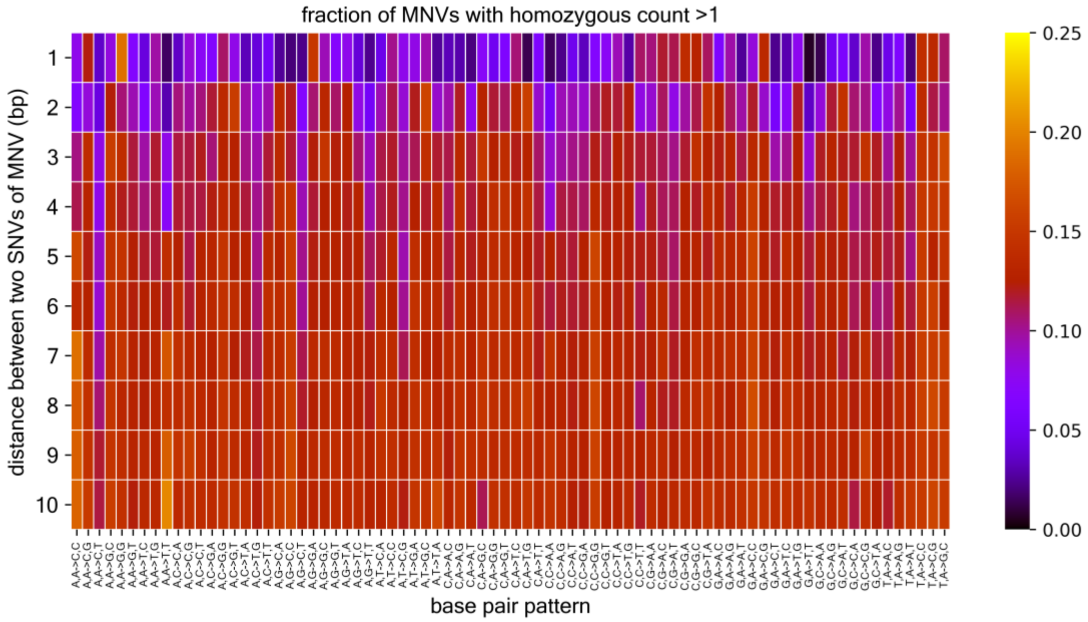

Supplementary Figure 13. Fraction of MNV that homozygous MNV are observed, up to 10 bp
Row denotes the distance between two SNVs of variant pairs, and column denotes the base pair pattern.

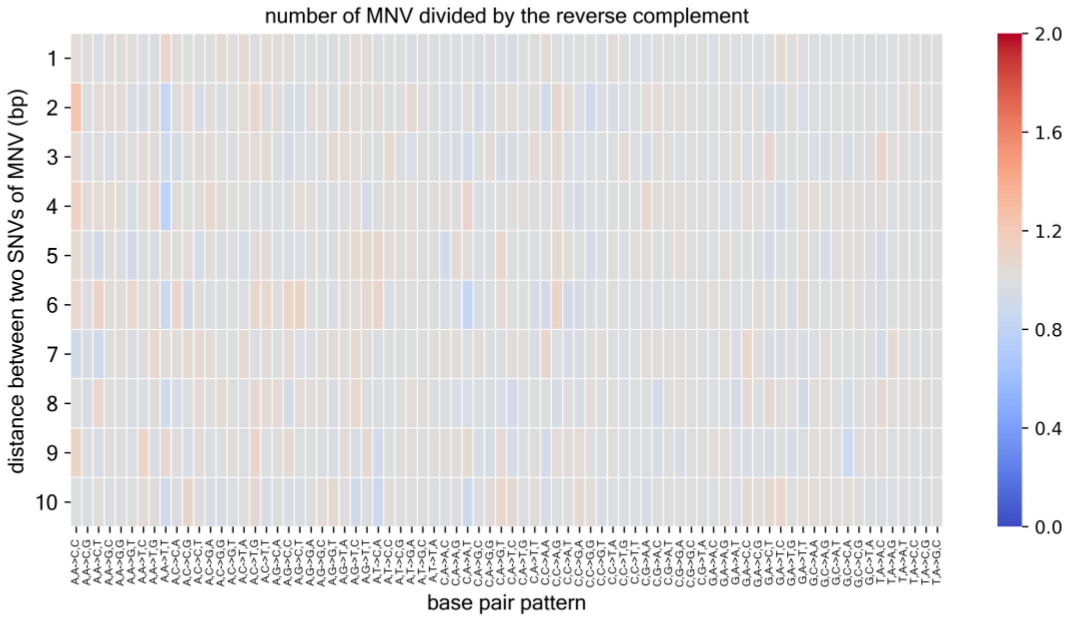

Supplementary Figure 14. ratio of number of MNV against their reverse complements, up to 10 bp
Row denotes the distance between two SNVs of variant pairs, and column denotes the base pair pattern. The ratio against their reverse complement is represented as color (color shifts in log scale).

Except for this figure, all the MNV pattern (d=1), and base pair pattern (d=1..10) do not distinguish the corresponding reverse complements.

a.

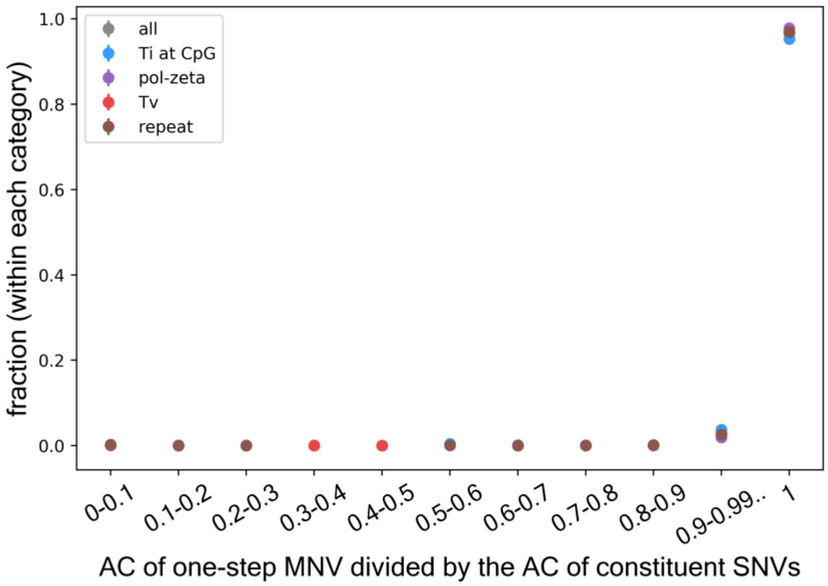

b.

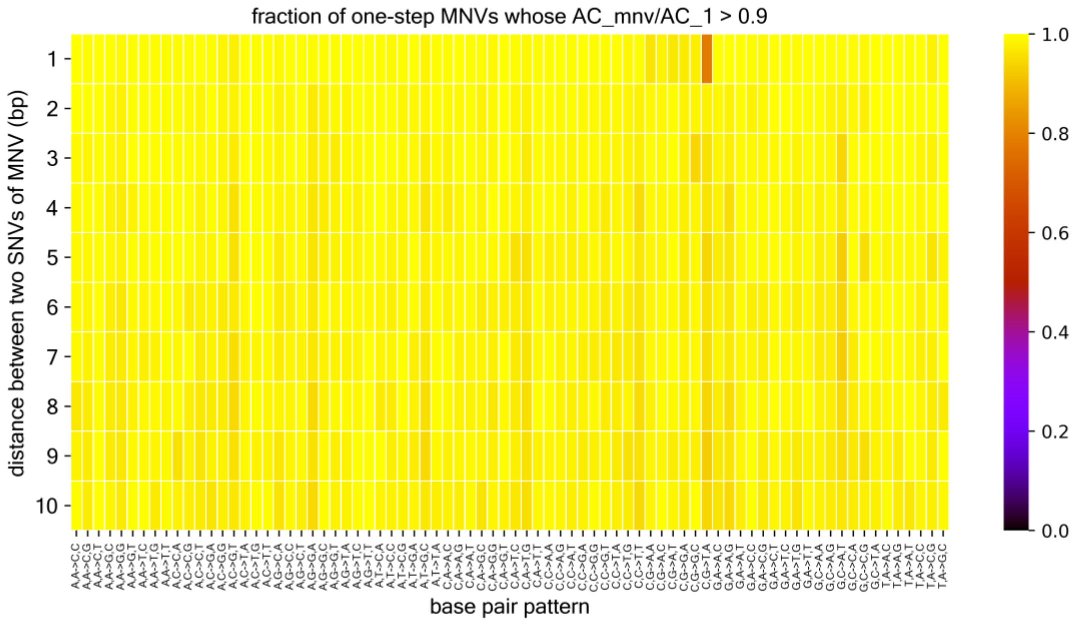

Supplementary Figure 15. Allele count of individual SNVs for one-step MNV
a, Distribution of allele count of one-step MNV divided by that of constituent SNVs**. b,** Fraction of one-step MNV whose AC_mnv is close enough (fraction > 0.9), up to 10 bp. Row denotes the distance

between two SNVs of variant pairs, and column denotes the base pair pattern. The fraction is represented as color. We took the threshold of AC_mnv / AC_1 >0.9 manually, after observing **(a)**.

a.

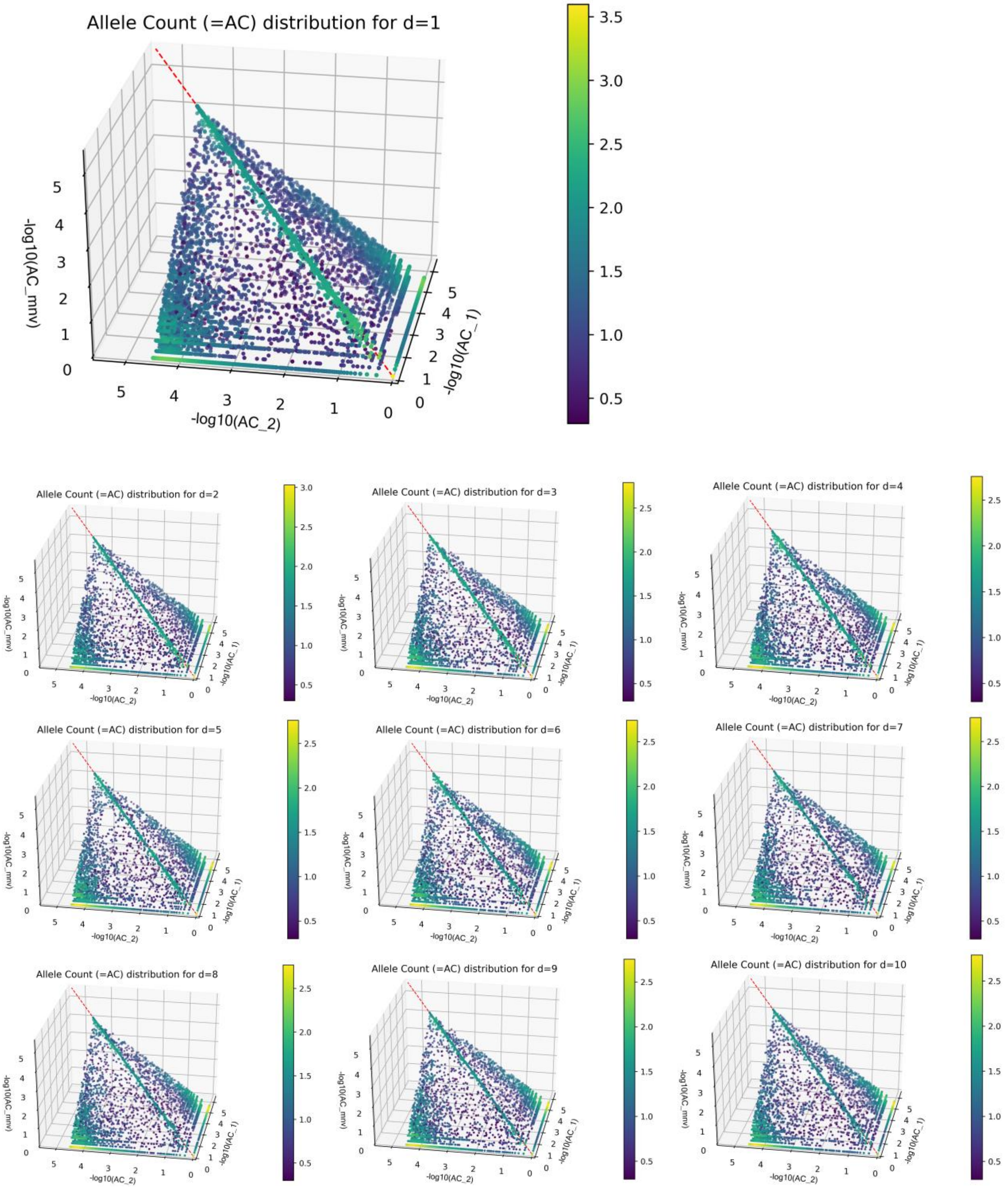

b.

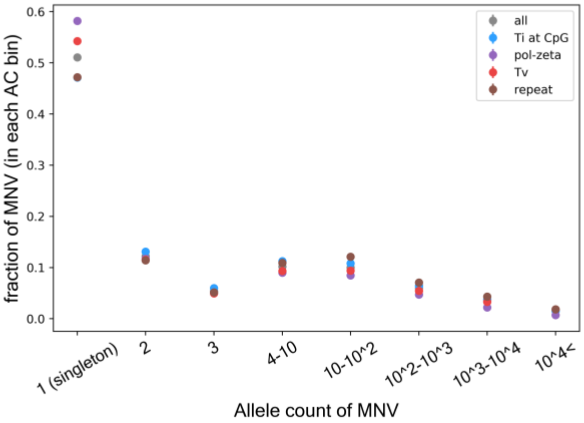

c.

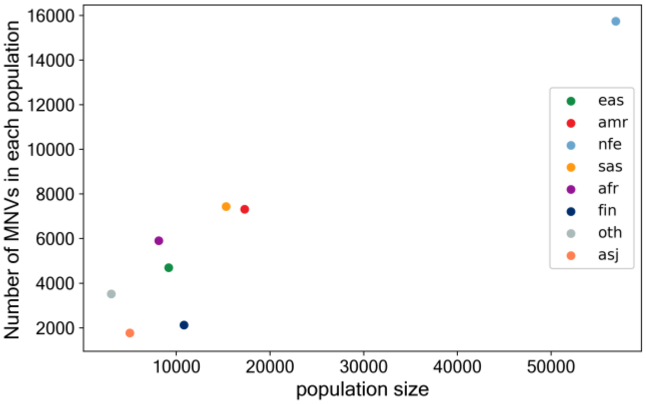

d.

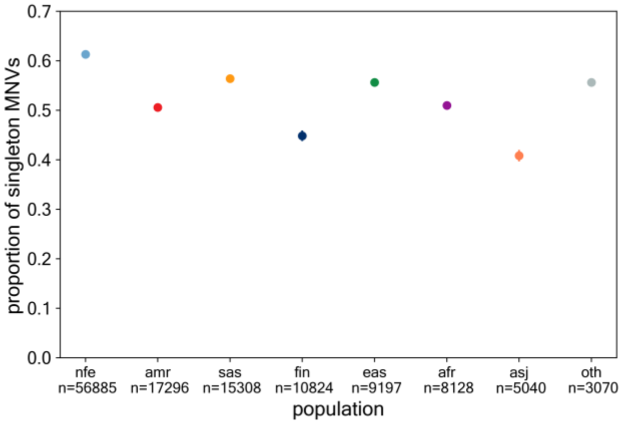

e.

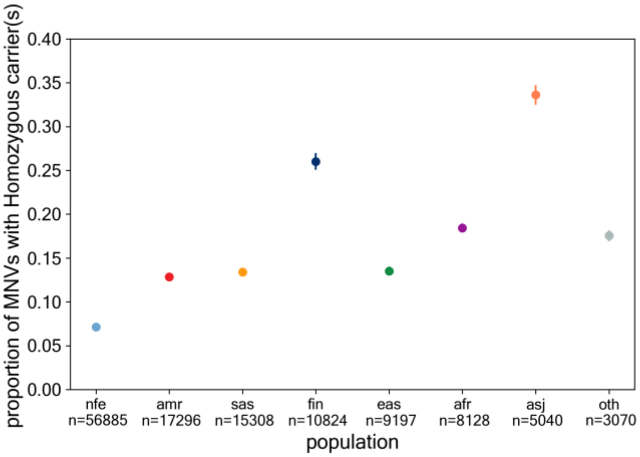

Supplementary Figure 16. Distribution of allele counts of MNVs, and comparison across populations

a, Overview of allele count of MNVs. One axis is the allele count of SNV that are upstream (AC_1), another for the allele count of SNV that are downstream (AC_2) in the reference genome, and the other axis is for the allele count of corresponding MNV (in log10 space). Color corresponds to the relative density (defined as the number of neighborhood counts in log-space), and the read dot line shows x=y=z (i.e. AC of SNV1 = AC of SNV2 = AC of MNV). Only the result of chr22 is shown, in order for the figure to be sparse enough for effective visualization. **b,** The allele count distribution for different adjacent MNV patterns characterized by different potential mechanisms. Proportion of singleton for Ti at CpG (0.471) is nearly identical to that of repeat (0.472). **c,** The sample size and the number of MNV discovered for each population. **d, e,** The proportion of singleton MNVs **(d)** and the proportion of MNVs in which homozygous carrier(s) are observed **(e)** within each population. nfe=Europian (non-Finnish), amr=Latino, sas=South Asian, fin=Europian (Finnish), eas=East Asian, afr=African, asj=Ashkenazi Jewish, oth=Others, ordered by the population size, and restricted to gnomAD exome data set.

a.

b.

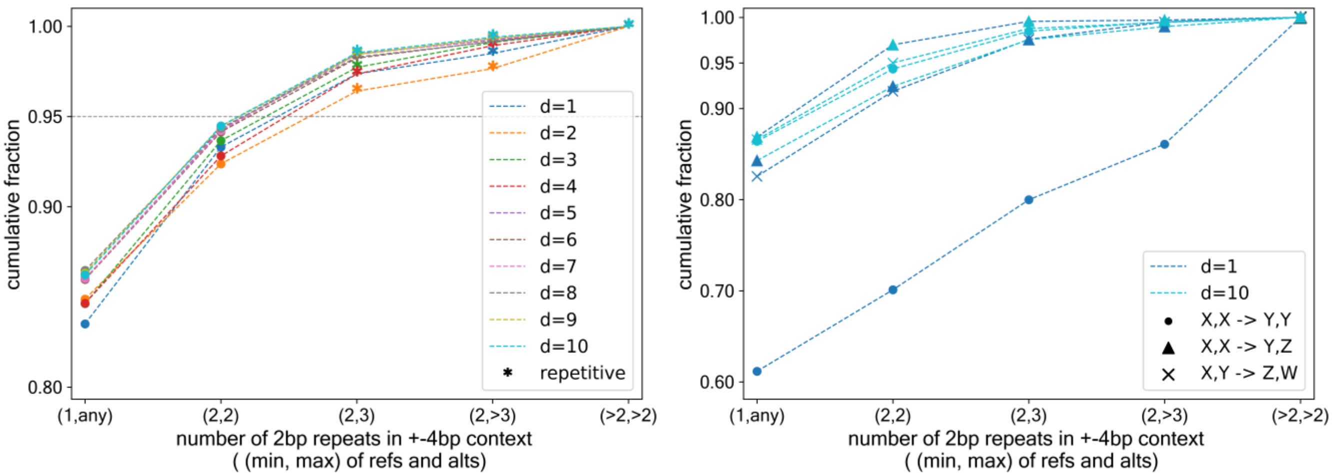

Supplementary Figure 17. Distribution of number of repeats
a, x axis is the maximum number of repeats (Unit = minimum and maximum of the reference +-4 bp and alternative +-4 bp context of the MNV.), and y is the cumulative density. Each line corresponds to specific distance between two SNVs of MNV, and the star shape shows that they pass the threshold of cumulative percentage >95%, and thus are defined as "repetitive". **b,** the distribution differences for different pattern of reference and alternative two bases (e.g. X,X->Y,Z means the first and second SNVs of the reference 2bp of the MNV are the same, but those of the alternative 2bp is different**)**. We corrected for the significant skew in the number of repeats in XX->YY type of adjacent MNVs when defining repetitive contexts, as described in the methods.

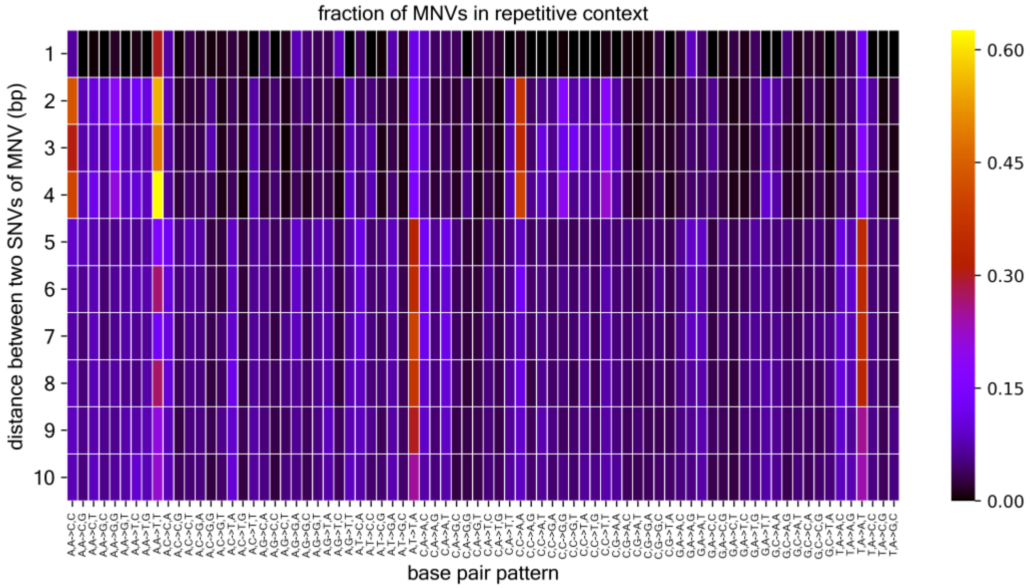

Supplementary Figure 18. Fraction of MNV that are in repetitive contexts, up to 10 bp
Row denotes the distance between two SNVs of variant pairs, and column denotes the base pair pattern. The fraction is represented as color.

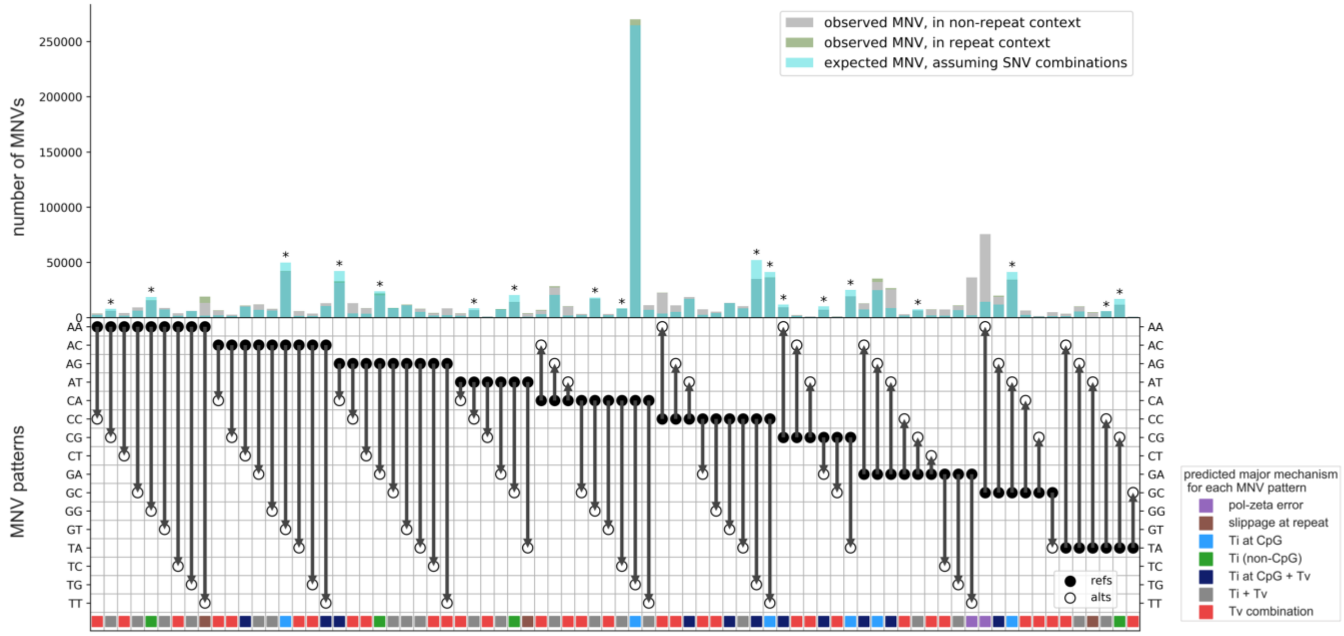

Supplementary Figure 19: Expected number of MNVs in the simulated model vs observed number of MNVs

Cyan shows the expected number of MNVs that originates from two SNV events, when assuming that all the MNVs of pattern CA->TG in non-repeat contexts originates from two SNV events. Green is the observed number of MNVs in repeat contexts, and grey is the observed number of MNVs in non-repeat contexts. The asterisk on top of the bar indicates that the estimated number of MNVs exceeds the observed (The level of overestimation was 1.48-fold at most, for the CC->TG MNVs). The color in the bottom is as explained in figure 4.

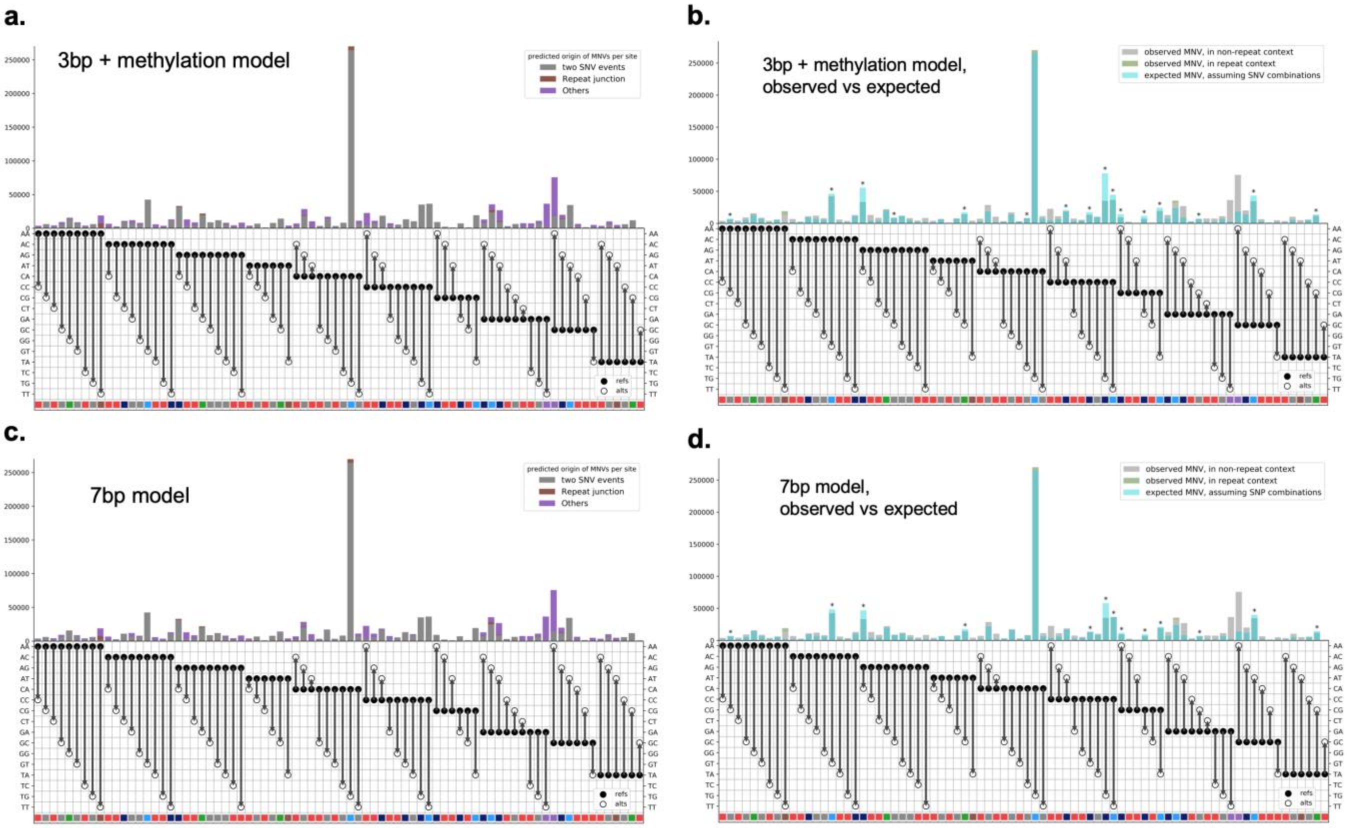

Supplementary Figure 20: MNVs in the simulated model vs observed number of MNVs, under different models

a, Estimated fraction of MNVs per different biological origin, when using the methylation model. **b,** Expected number of MNVs that originates from two SNV events vs observed number of MNVs, when using methylation model. The level of overestimation for the CC->TG MNVs was 2.05-fold, higher than the canonical model. **c,** Estimated fraction of MNVs per different biological origin, when using the 7 bp context model. **d,** Expected number of MNVs that originates from two SNV events vs observed number of MNVs, when using the 7 bp context model. The level of overestimation for the CC->TG MNVs was 1.54-fold, higher than the canonical model. Axis and the color code are as explained in **figure 4** for **(a)** and **(c)**, and are as explained in the **figure S19** for **(b)** and **(d)**.

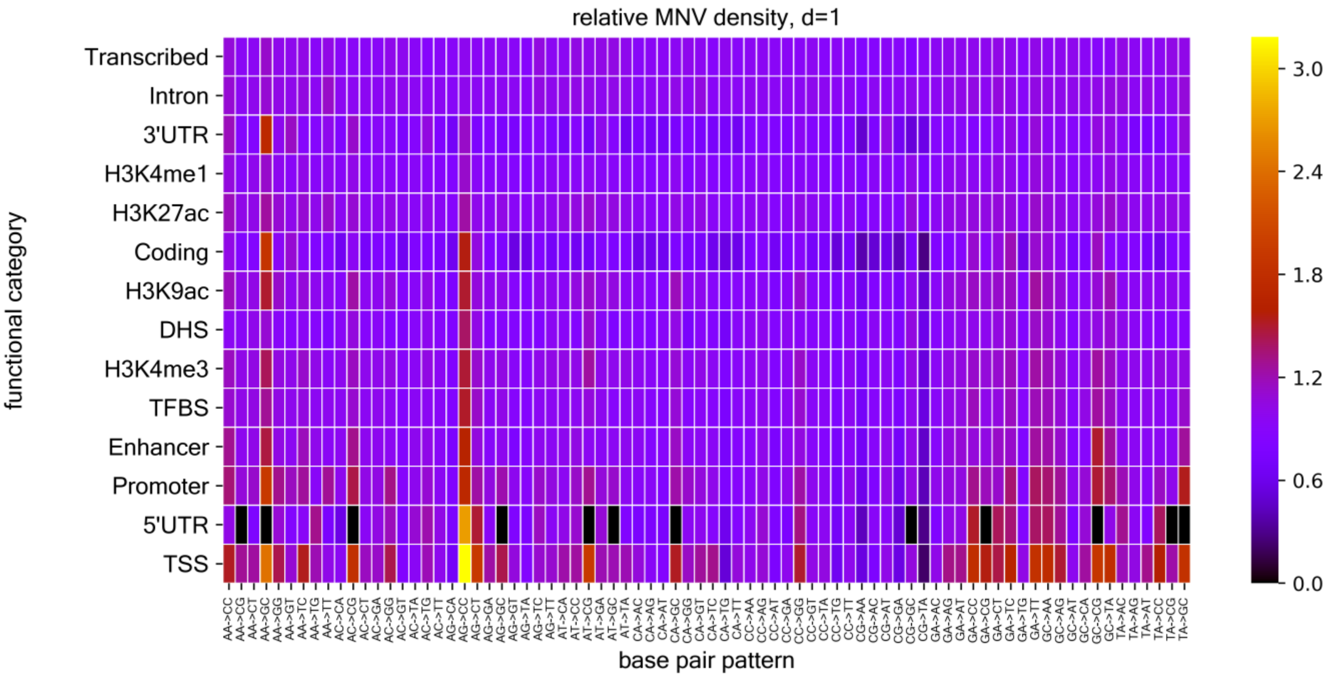
relative MNV density, d=1
Transcribed
Intron
3'UTR
H3K4me1
H3K27ac
Coding
H3K9ac
DHS
H3K4me3
TFBS
Enhancer
Promoter
5'UTR
TSS
functional category
base pair pattern
3.0
2.4
1.8
1.2
0.6
0.0

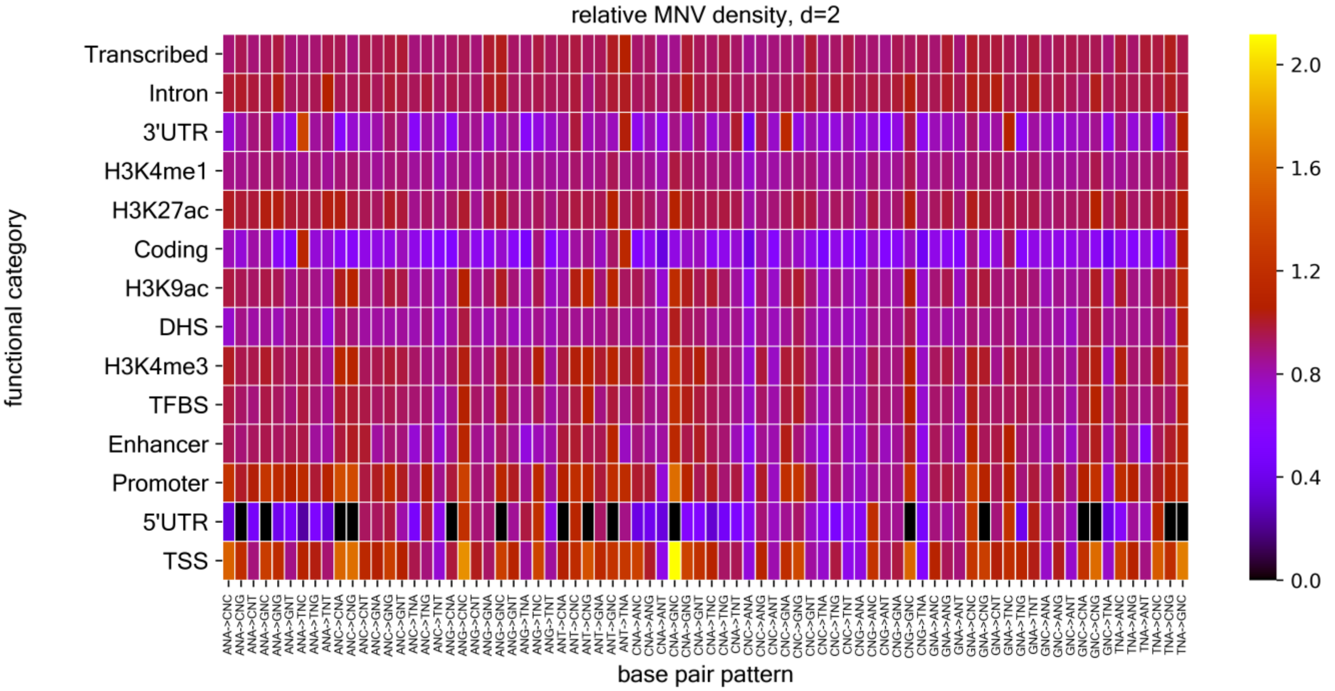
relative MNV density, d=2
Transcribed
Intron
3'UTR
H3K4me1
H3K27ac
Coding
H3K9ac
DHS
H3K4me3
TFBS
Enhancer
Promoter
5'UTR
TSS
functional category
base pair pattern
2.0
1.6
1.2
0.8
0.4
0.0

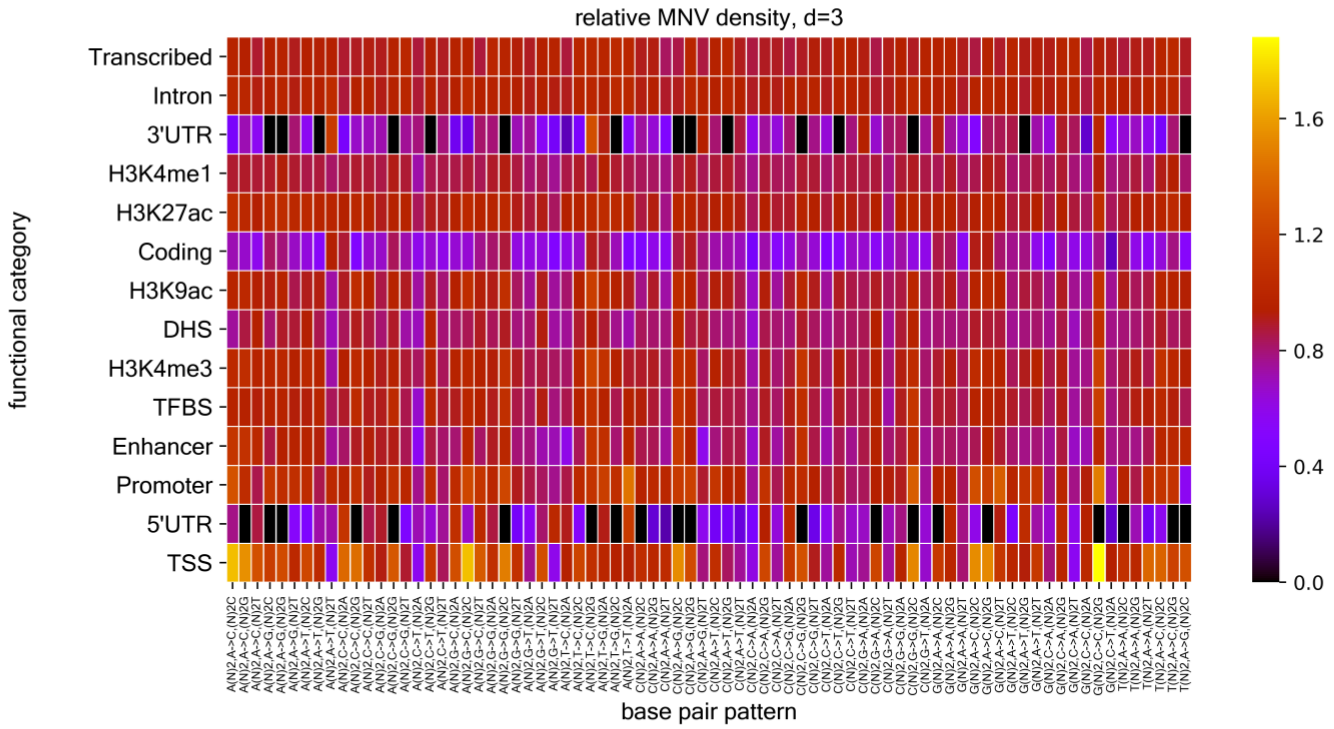
relative MNV density, d=3
functional category
Transcribed
Intron
3'UTR
H3K4me1
H3K27ac
Coding
H3K9ac
DHS
H3K4me3
TFBS
Enhancer
Promoter
5'UTR
TSS
base pair pattern
1.6
1.2
0.8
0.4
0.0

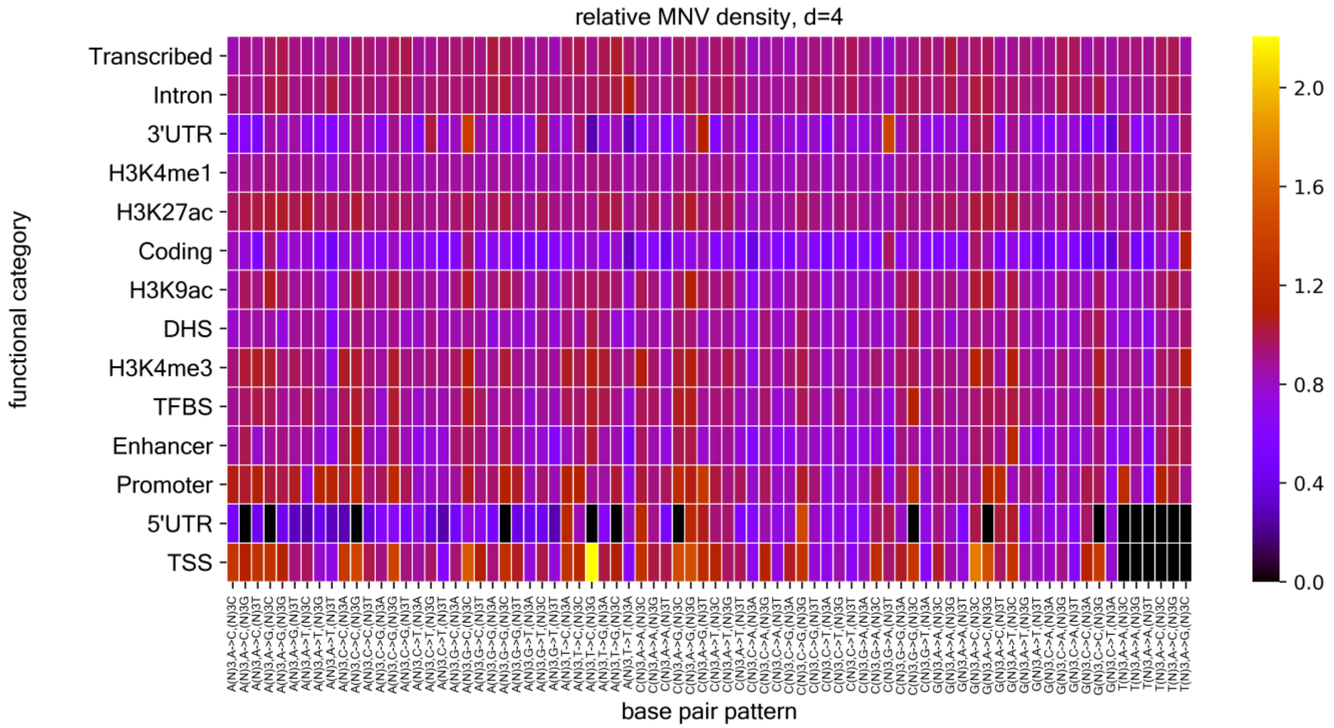
relative MNV density, d=4
functional category
Transcribed
Intron
3'UTR
H3K4me1
H3K27ac
Coding
H3K9ac
DHS
H3K4me3
TFBS
Enhancer
Promoter
5'UTR
TSS
base pair pattern
2.0
1.6
1.2
0.8
0.4
0.0

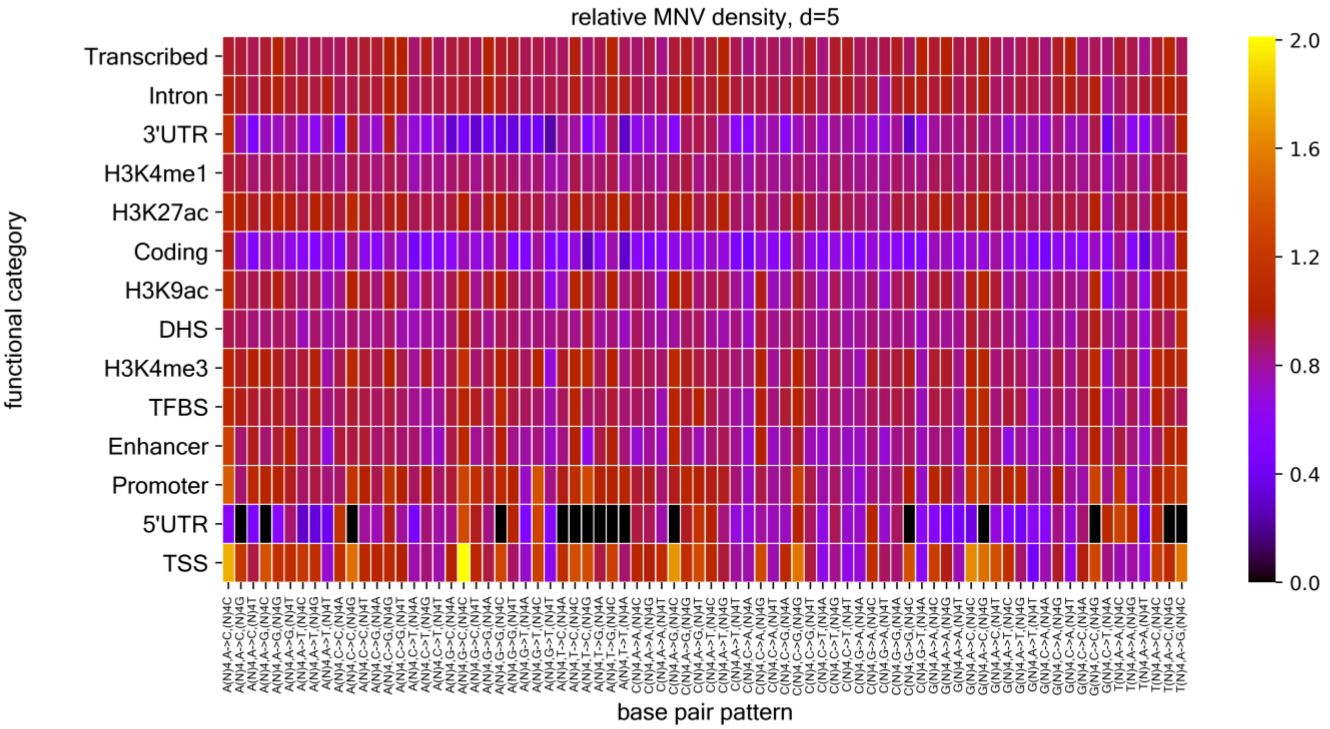
relative MNV density, d=5
functional category
Transcribed
Intron
3'UTR
H3K4me1
H3K27ac
Coding
H3K9ac
DHS
H3K4me3
TFBS
Enhancer
Promoter
5'UTR
TSS
base pair pattern
2.0
1.6
1.2
0.8
0.4
0.0

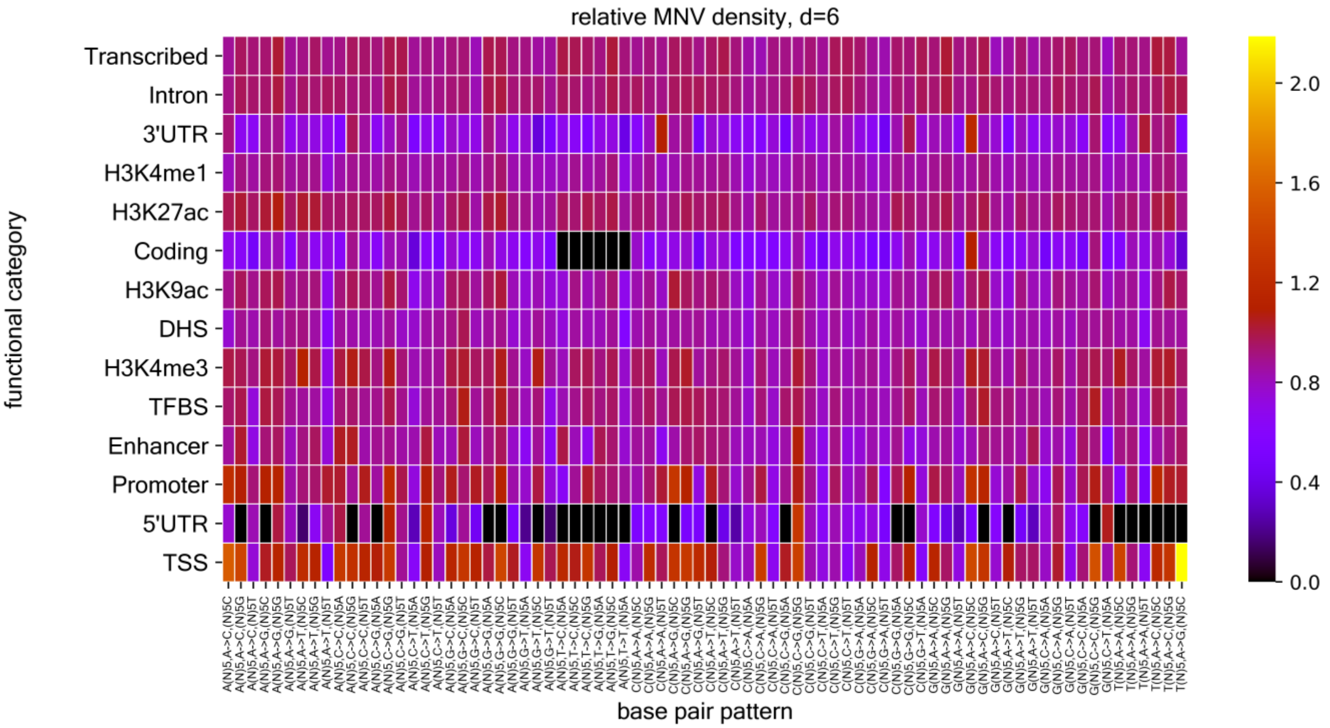
relative MNV density, d=6
functional category
Transcribed
Intron
3'UTR
H3K4me1
H3K27ac
Coding
H3K9ac
DHS
H3K4me3
TFBS
Enhancer
Promoter
5'UTR
TSS
base pair pattern
2.0
1.6
1.2
0.8
0.4
0.0

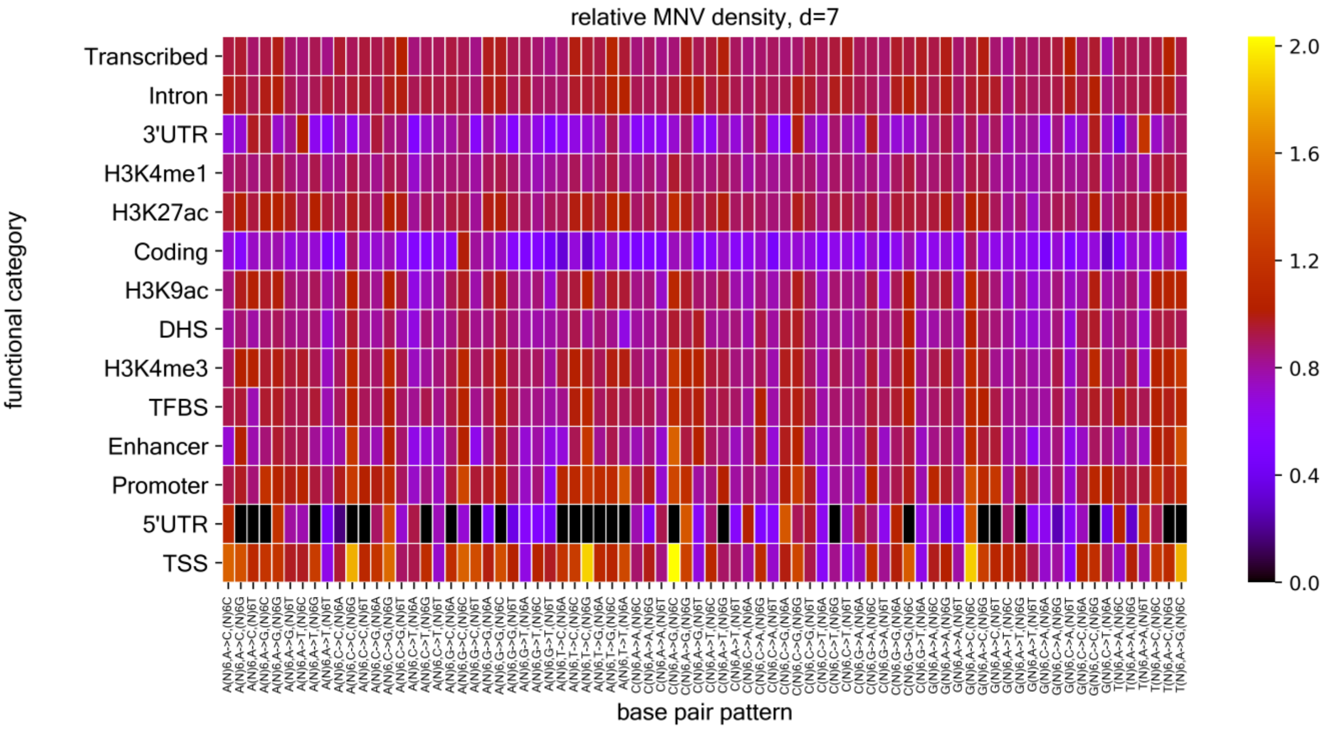
relative MNV density, d=7
Transcribed
Intron
3'UTR
H3K4me1
H3K27ac
Coding
H3K9ac
DHS
H3K4me3
TFBS
Enhancer
Promoter
5'UTR
TSS
functional category
base pair pattern
2.0
1.6
1.2
0.8
0.4
0.0

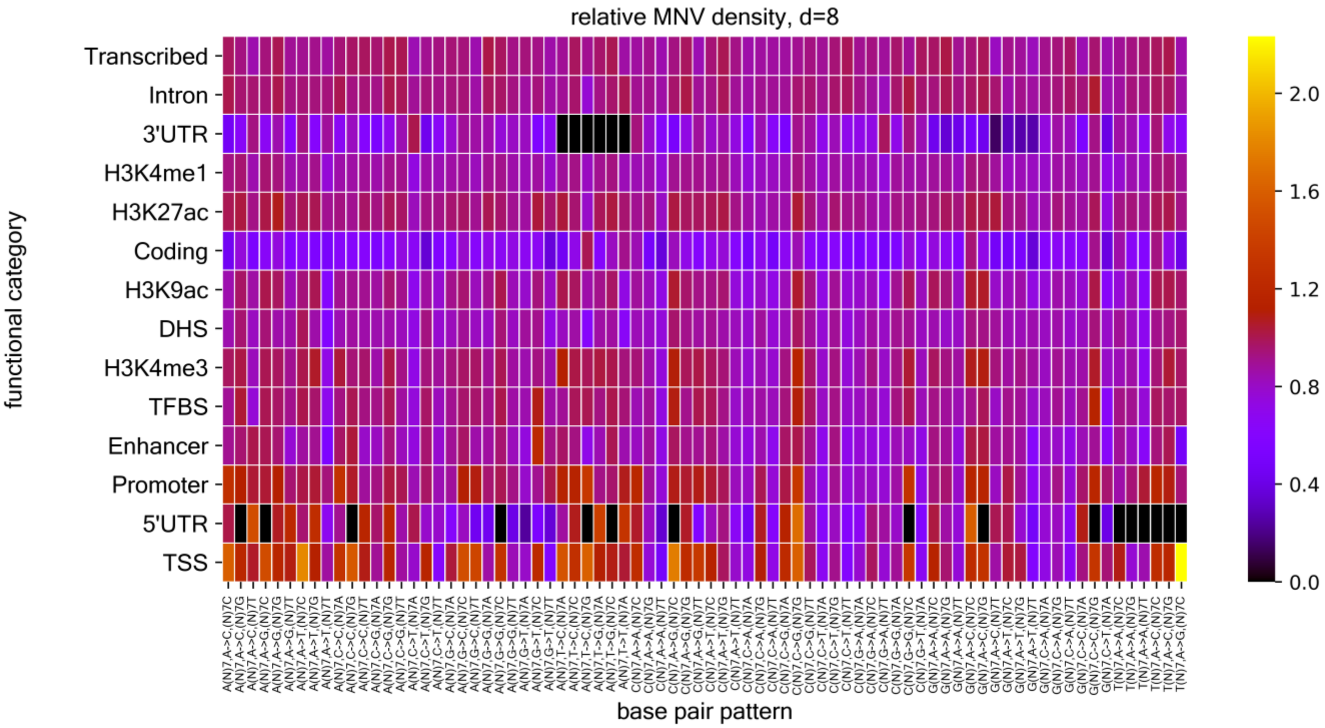
relative MNV density, d=8
Transcribed
Intron
3'UTR
H3K4me1
H3K27ac
Coding
H3K9ac
DHS
H3K4me3
TFBS
Enhancer
Promoter
5'UTR
TSS
functional category
base pair pattern
2.0
1.6
1.2
0.8
0.4
0.0

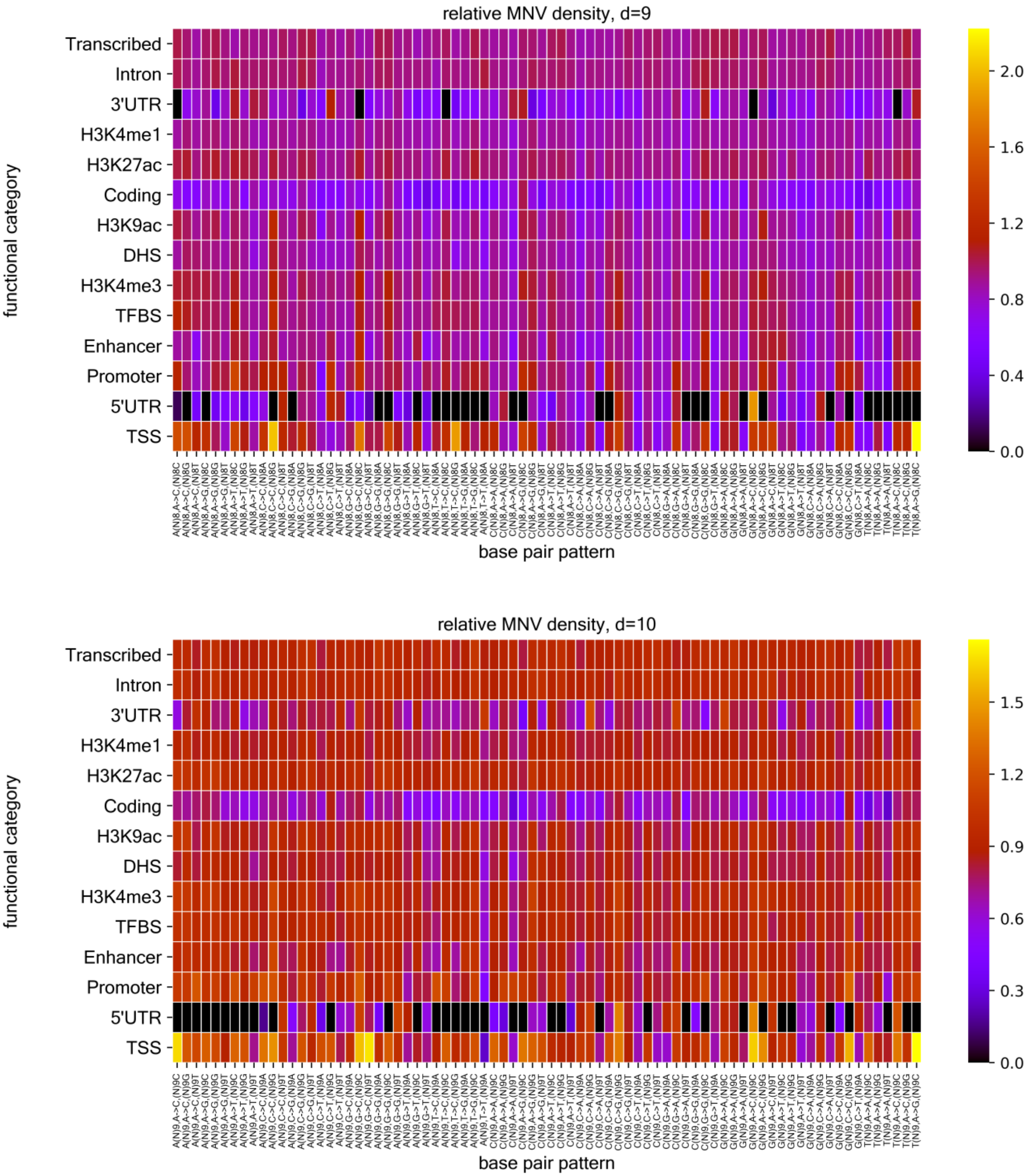

Supplementary Figure 21. Relative MNV density per functional category per MNV pattern
Row denotes each functional category, descending order from the top by average methylation level, and column denotes the base pair pattern. The fraction is represented as color. For some of the functional category such as UTRs, the overall count is extremely low, resulting in often 0 density.
