# Supplementary material for: Landscape of multi-nucleotide variants in 125,748 human exomes and 15,708 genomes
Source: Nat Commun. 2020 May 27;11:2539. doi: 10.1038/s41467-019-12438-5 (PMC7253413; doi:10.1038/s41467-019-12438-5)
Supplement: Supplementary file 6 — Description of Additional Supplementary Files [file 41467_2019_12438_MOESM6_ESM.pdf]

Title: Supplementary Data 1.
Description: Definition of MNV functional category classification and counts in gnomAD exome, list of genes with more than zero gained nonsense mutation or rescued nonsense mutation, and the full list of MNV count per gene per category

Title: Supplementary Data 2.
Description: List of gained nonsense mutation, changed and gained missense with high CADD score, observed in rare disease samples, with a brief phenotype breakdown of the samples.

Title: Supplementary Data 3.
Description: Estimation of MNV frequency per MNV pattern per generation, and the breakdown of predicted major mechanism for each MNV pattern.
