# Supplementary material for: Landscape of multi-nucleotide variants in 125,748 human exomes and 15,708 genomes
Source: Nat Commun. 2020 May 27;11:2539. doi: 10.1038/s41467-019-12438-5 (PMC7253413; doi:10.1038/s41467-019-12438-5)
Supplement: Supplementary file 7 — Reporting Summary [file 41467_2019_12438_MOESM7_ESM.pdf]

nature research

Corresponding author(s): Qingbo Wang / Daniel MacArthur

Last updated by author(s): Sep 4, 2019

# Reporting Summary

Nature Research wishes to improve the reproducibility of the work that we publish. This form provides structure for consistency and transparency in reporting. For further information on Nature Research policies, see Authors & Referees and the Editorial Policy Checklist.

## Statistics

For all statistical analyses, confirm that the following items are present in the figure legend, table legend, main text, or Methods section.

| n/a | Confirmed | |
|---|---|---|
| ☐ | ☒ | The exact sample size (*n*) for each experimental group/condition, given as a discrete number and unit of measurement |
| ☒ | ☐ | A statement on whether measurements were taken from distinct samples or whether the same sample was measured repeatedly |
| ☐ | ☒ | The statistical test(s) used AND whether they are one- or two-sided<br>*Only common tests should be described solely by name; describe more complex techniques in the Methods section.* |
| ☐ | ☒ | A description of all covariates tested |
| ☐ | ☒ | A description of any assumptions or corrections, such as tests of normality and adjustment for multiple comparisons |
| ☐ | ☒ | A full description of the statistical parameters including central tendency (e.g. means) or other basic estimates (e.g. regression coefficient) AND variation (e.g. standard deviation) or associated estimates of uncertainty (e.g. confidence intervals) |
| ☐ | ☒ | For null hypothesis testing, the test statistic (e.g. *F*, *t*, *r*) with confidence intervals, effect sizes, degrees of freedom and *P* value noted<br>*Give P values as exact values whenever suitable.* |
| ☒ | ☐ | For Bayesian analysis, information on the choice of priors and Markov chain Monte Carlo settings |
| ☒ | ☐ | For hierarchical and complex designs, identification of the appropriate level for tests and full reporting of outcomes |
| ☐ | ☒ | Estimates of effect sizes (e.g. Cohen's *d*, Pearson's *r*), indicating how they were calculated |

*Our web collection on statistics for biologists contains articles on many of the points above.*

## Software and code

Policy information about availability of computer code

| | |
|---|---|
| Data collection | We used Genome Analysis Toolkit https://software.broadinstitute.org/gatk/ for variant discovery and genotyping. |
| Data analysis | We used Hail http://hail.is/ for the analysis.<br>The code used in the study is available at https://github.com/macarthur-lab/gnomad_mnv. |

For manuscripts utilizing custom algorithms or software that are central to the research but not yet described in published literature, software must be made available to editors/reviewers. We strongly encourage code deposition in a community repository (e.g. GitHub). See the Nature Research guidelines for submitting code & software for further information.

## Data

Policy information about availability of data

All manuscripts must include a data availability statement. This statement should provide the following information, where applicable:
- Accession codes, unique identifiers, or web links for publicly available datasets
- A list of figures that have associated raw data
- A description of any restrictions on data availability

The list of coding MNVs in gnomAD exome are available at gs://gnomad-public/release/2.1/mnv/gnomad_mnv_coding.tsv (tab separated file). The coding MNVs consisting of three SNVs in a single codon is available as a separate file at gs://gnomad-public/release/2.1/mnv/gnomad_mnv_coding_3bp.tsv .
The list of frame-restoring indel pairs are available at gs://gnomad-public/release/2.1/mnv/frame_restoring_indels.tsv.
The list of all the MNVs in gnomAD genomes are available at gs://gnomad-public/release/2.1/mnv/genome/gnomad_mnv_genome_d{i}.tsv.bgz (tab separated file, compressed. Replace {i} (0<i<11) with the distance between two SNVs of MNV.), or gs://gnomad-public/release/2.1/mnv/genome/gnomad_mnv_genome_d{i}.ht (hail table. Replace {i} (0<i<11) with the distance between two SNVs of MNV.).





Explanations for each column in each file can be found at gs://gnomad-public/release/2.1/mnv/mnv_readme.md .
All the files above are also available at the download page of the gnomAD browser (https://gnomad.broadinstitute.org/downloads).

# Field-specific reporting

Please select the one below that is the best fit for your research. If you are not sure, read the appropriate sections before making your selection.

☒ Life sciences ☐ Behavioural & social sciences ☐ Ecological, evolutionary & environmental sciences

For a reference copy of the document with all sections, see nature.com/documents/nr-reporting-summary-flat.pdf

# Life sciences study design

All studies must disclose on these points even when the disclosure is negative.

| | |
|---|---|
| Sample size | NA |
| Data exclusions | NA |
| Replication | NA |
| Randomization | NA |
| Blinding | NA |

# Reporting for specific materials, systems and methods

We require information from authors about some types of materials, experimental systems and methods used in many studies. Here, indicate whether each material, system or method listed is relevant to your study. If you are not sure if a list item applies to your research, read the appropriate section before selecting a response.

## Materials & experimental systems

| n/a | Involved in the study | |
|---|---|---|
| ☒ | ☐ | Antibodies |
| ☒ | ☐ | Eukaryotic cell lines |
| ☒ | ☐ | Palaeontology |
| ☒ | ☐ | Animals and other organisms |
| ☒ | ☐ | Human research participants |
| ☒ | ☐ | Clinical data |

## Methods

| n/a | Involved in the study | |
|---|---|---|
| ☒ | ☐ | ChIP-seq |
| ☒ | ☐ | Flow cytometry |
| ☒ | ☐ | MRI-based neuroimaging |
